# A Computational Model of the Endothelial to Mesenchymal Transition

**DOI:** 10.3389/fgene.2020.00040

**Published:** 2020-03-12

**Authors:** Nathan Weinstein, Luis Mendoza, Elena R. Álvarez-Buylla

**Affiliations:** ^1^Instituto de Ecología, Universidad Nacional Autónoma de México, Mexico City, Mexico; ^2^Instituto de Investigaciones Biomédicas, Universidad Nacional Autónoma de México, Mexico City, Mexico; ^3^Centro de Ciencias de la Complejidad, Universidad Nacional Autónoma de México, Mexico City, Mexico

**Keywords:** endothelial-mesenchymal transition, systems biology, angiogenesis, Boolean network, endothelial cell plasticity, heart development, fibrosis

## Abstract

Endothelial cells (ECs) form the lining of lymph and blood vessels. Changes in tissue requirements or wounds may cause ECs to behave as tip or stalk cells. Alternatively, they may differentiate into mesenchymal cells (MCs). These processes are known as EC activation and endothelial-to-mesenchymal transition (EndMT), respectively. EndMT, Tip, and Stalk EC behaviors all require SNAI1, SNAI2, and Matrix metallopeptidase (MMP) function. However, only EndMT inhibits the expression of VE-cadherin, PECAM1, and VEGFR2, and also leads to EC detachment. Physiologically, EndMT is involved in heart valve development, while a defective EndMT regulation is involved in the physiopathology of cardiovascular malformations, congenital heart disease, systemic and organ fibrosis, pulmonary arterial hypertension, and atherosclerosis. Therefore, the control of EndMT has many promising potential applications in regenerative medicine. Despite the fact that many molecular components involved in EC activation and EndMT have been characterized, the system-level molecular mechanisms involved in this process have not been elucidated. Toward this end, hereby we present Boolean network model of the molecular involved in the regulation of EC activation and EndMT. The simulated dynamic behavior of our model reaches fixed and cyclic patterns of activation that correspond to the expected EC and MC cell types and behaviors, recovering most of the specific effects of simple gain and loss-of-function mutations as well as the conditions associated with the progression of several diseases. Therefore, our model constitutes a theoretical framework that can be used to generate hypotheses and guide experimental inquiry to comprehend the regulatory mechanisms behind EndMT. Our main findings include that both the extracellular microevironment and the pattern of molecular activity within the cell regulate EndMT. EndMT requires a lack of VEGFA and sufficient oxygen in the extracellular microenvironment as well as no FLI1 and GATA2 activity within the cell. Additionally Tip cells cannot undergo EndMT directly. Furthermore, the specific conditions that are sufficient to trigger EndMT depend on the specific pattern of molecular activation within the cell.

## Introduction

The circulatory system allows the body to efficiently transport oxygen and nutrients to all the constituent cells of animals through an intrincate network of blood vessels. Capillaries are the smallest blood vessels, communicating arterioles and venules; they are composed of a single layer of endothelial cells (ECs), and are partially covered by mural cells called pericytes (PCs). ECs and PCs are in close proximity to most cells in multicellular animals and are some of the most important cells involved in wound healing and tissue regeneration. Thus, alterations that affect these cells result in several pathological processes (Eming et al., 2014; Birbrair et al., 2015).

**Figure 1:**
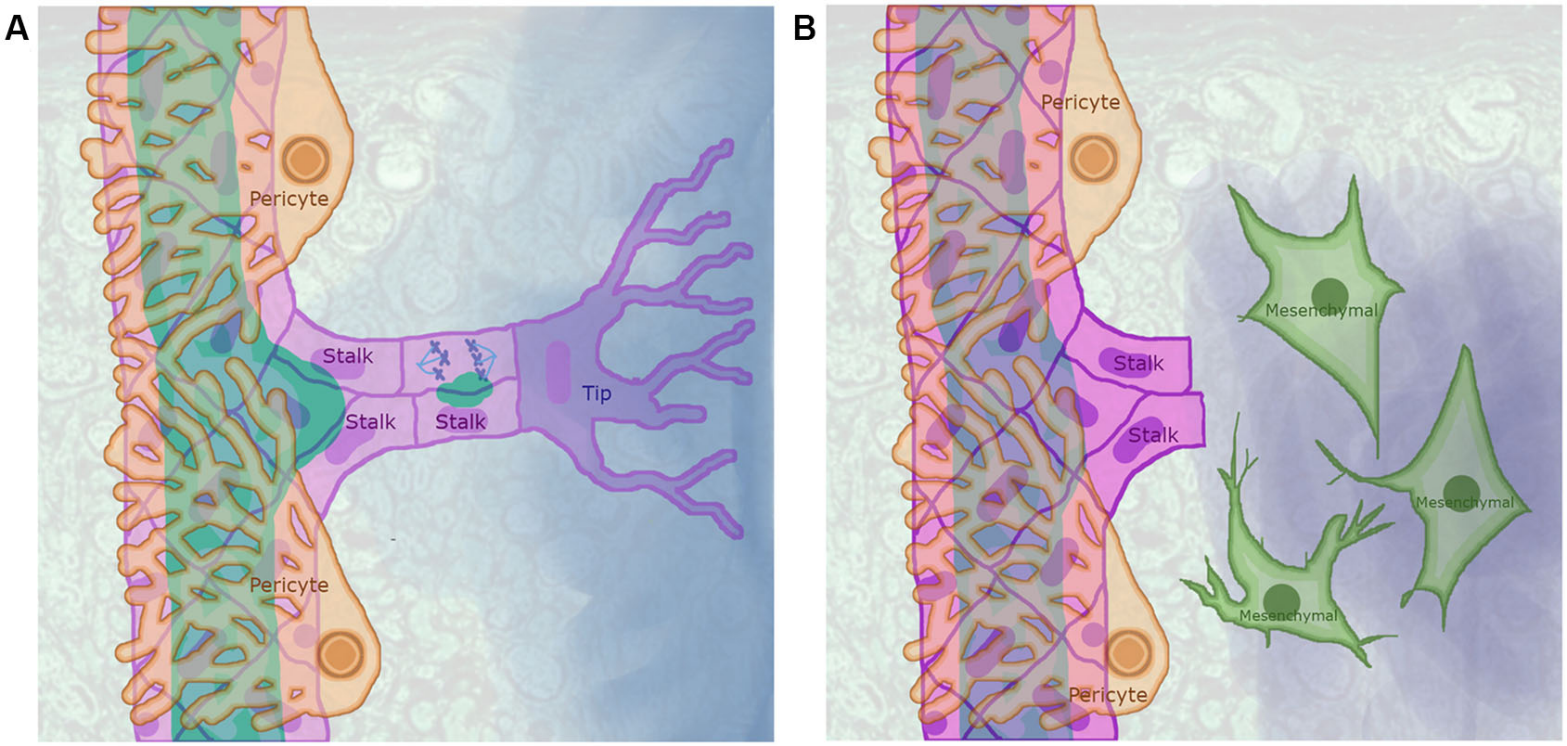
Sprouting angiogenesis as partial endothelial-to-mesenchymal transition (EndMT): **(A)** In a precapillary arteriole with an angiogenic sprout, the pericytes (light orange cells that surround the arteriole) detach from a region of the arteriole exposed to a concentration of angiogenic signal that exceeded a certain threshold leading to the activation of an endothelial cell (EC) that became a Tip cell (purple) that extends filipodia to sense the angiogenic signal gradient. The ECs that surrounded the Tip cell where induced to become Stalk cells (pink) that proliferate, elongate, secrete vacuoles, and trail the tip cell as it migrates following the angiogenic signal gradient. **(B)** The EndMT process is similar to sprouting angiogenesis, as ECs have to be activated and secrete Matrix metallopeptidases that degrade the basement membrane to increase their motility and proliferate. However, in contrast to Tip and Stalk cells, ECs that undergo EndMT completely detach from other ECs and stop expressing EC markers.

While ECs and PCs are fully differentiated cell types, they have the notable capacity to trans-differentiate into each other (Nakagomi et al., 2015; Chen et al., 2016; Jackson et al., 2017), and are also capable of differentiating into hematopoietic stem cells, mesenchymal stem cells, and several other cell types (van Meeteren and Ten Dijke, 2012; Birbrair et al., 2017; Dejana et al., 2017). Notably, ECs differentiate into PCs in a process called endothelial to mesenchymal transition (EndMT), which is very similar to the epithelial-to-mesenchymal transition (EMT) (Lamouille et al., 2014; Méndez-López et al., 2017). Like EMT, EndMT is a reversible process, and the opposite mechanism is denominated mesenchymal-to-endothelial transition (MEnT) (Sánchez-Duffhues et al., 2018). EndMT is triggered either by changes in the concentration of WNT, NOTCH, FGF, or TGF ligands in the extracellular microenvironment, reduced oxygen availability or shear stress. These changes lead to the activation of the transcription factors SNAI1, SNAI2, TWIST1, ZEB1, and SPI1(ZEB2), resulting in the repression of the expression of endothelial markers, specifically VEGFR2, PECAM1, VE-Cadherin, TIE1, TIE2, and vWF accompanied by the augmented expression of mesenchymal markers including α-SMA, N-cadherin, and Collagen I//II. During EndMT, ECs lose cell-to-cell adhesion and luminobasal polarity, gaining migratory and invasive potential (**Figure 1B**) (Gong et al., 2017; Jackson et al., 2017).

EndMT is a key process; physiologically, it is present during the development of the heart. The formation and maturation of the endocardial cushion leads to the formation of the septa and valves. First, the endocardial cells located at the atrioventricular canal (AVC)—including endocardial ECs—separate from the myocardial cells that cover them. Then, the endocardial and myocardial cells secrete extracellular matrix (ECM) components that accumulate to form and expand the cardiac matrix jelly that separates them. After that, AVC myocardial cells secrete bone morphogenetic proteins (BMPs), causing AVC ECs to undergo EndMT. Lastly, the mesenchymal cells resulting from the EndMT differentiate into the cells that compose the cardiac septa and heart valves (Kaneko et al., 2008). From the pathological perspective, EndMT alterations are involved in many cardiovascular disorders including artherosclerosis, congenital heart disease, myocardial fibrosis, myocardial infractions, and pulmonary arterial hypertension.

Stable vascular networks are lined by a layer of quiescent ECs called *Phalanx cells* that are tightly bound to each other and to the basement membrane, as well as being at least partially covered by PCs. These Phalanx ECs do not proliferate, however, they do exhibit lumen to basal membrane polarity, and express EC markers (Korn and Augustin, 2015; Betz et al., 2016). Either hypoxia or the lack of sufficient nutrients may cause cells that surround a microvascular network to secrete angiogenic factors, triggering sprouting angiogenesis. In this process, certain ECs are induced to become migratory, invasive *Tip cells* (TCs), while adjacent PCs detach from the capillary segment. Each TC induces abutting ECs to become *Stalk cells* (SCs). Then, both the TC and SCs detach from the basement membrane and the TC migrates toward the source of the angiogenic signal trailing SCs that elongate and proliferate (**Figure 1A**). The new sprout continues to grow until the TC reaches either another blood vessel or the TC leading another sprout. Then, the lumen of the new segment is formed from the fusion of vacuoles (Jianxin et al., 2015; Kim et al., 2017) and flow-mediated apical membrane invagination (Gebala et al., 2016). Lastly, the new capillary segment is stabilized and surrounded by PCs.

During sprouting angiogenesis TCs and SCs detach from the basement membrane, migrate, and lose their luminobasal polarity. Furthermore, TCs are invasive and secrete MMPs that degrade the ECM while SCs proliferate. However, during angiogenesis, ECs continue to express their characteristic molecular markers, and the adherens and tight junctions that bind ECs remain intact, thus suggesting that TC and SC behavior involves partial EndMT (Welch-Reardon et al., 2015). Both TCs and SCs express SNAI1 and SNAI2, and silencing either of these genes inhibits angiogenic sprout formation, TC migration, and affects lumen formation. SNAI2 directly regulates the expression of MT1-MMP, the protein encoded by this gene cleaves and activates MMP2 and MMP9. These are two proteases involved in ECM degradation during sprouting angiogenesis (Welch-Reardon et al., 2014).

As summarized above, a large set of molecules has been described to be involved in angiogenesis and EndMT. Nonetheless, the integrated dynamical mechanisms that underlie full or partial EndMT are still not well understood (Welch-Reardon et al., 2015). We propose that theoretical and system-biology approaches, such as those proposed by (Álvarez-Buylla Roces et al., 2018; Yang and Albert, 2019), can help us elucidate the molecular mechanisms involved in EndMT regulation. Cell types and behaviors are defined by a combination of morphological, behavioral, genetic, and epigenetic traits (Pavillon and Smith, 2019). In molecular regulatory network models, cell types and behaviors are represented by fixed and cyclic patterns of molecular activation called attractors. Both ECs and MCs are very diverse groups of cells with different developmental origins and exhibit many patterns of gene expression and molecular activation (Chi et al., 2003; Ho et al., 2018) Therefore, we expect the underlying molecular mechanism involved in EC and MC identity and behavior regulation to be multistable.

Due to the enormous biological and medical importance of angiogenesis and EMT, both processes have been widely explored through the simulation of models at the molecular and cellular levels (Peirce, 2008; Qutub et al., 2009; Lu et al., 2013; Steinway et al., 2014; Heck et al., 2015; Li et al., 2016; Méndez-López et al., 2017; Weinstein et al., 2017; Suzuki et al., 2018). In contrast, to the best of our knowledge, simulation or formal analyses of the molecular mechanism that control EndMT are lacking. To this aim, we inferred the regulatory network of EndMT by undertaking an exhaustive search of published data, and formalizing it as a dynamical network system to study its behavior under wild type and mutant backgrounds. The model is able to recover the expression patterns that characterize the main cell types during normal and pathological angiogenesis. Importantly, the model can be used as a tool to generate hypotheses regarding molecular and cellular effects of a large group of perturbations, such as mutations and pharmacological manipulations. Our main findings are that the specific conditions sufficient to trigger EndMT and MEnT depend on the pattern of molecular activation within the cell. EndMT requires a lack of FLI1 and GATA2 activity within the cell and also requires the absence of VEGFA and the presence of sufficient oxygen in the extracellular microenvironment. Additionally Tip cells cannot undergo EndMT directly.

## Methods

We assembled the molecular regulatory network of EndMT using information available in the literature, focusing on the incorporation of key molecules and their regulatory interactions. Then, the inferred network was transformed into a discrete dynamical system in the form of a Boolean network (BN). We analyzed the dynamical behavior of the model to find and classify the stationary and cyclic patterns of molecular activation. Thereafter, we studied the conditions that led to changes in the behavior or identity of the cells. Also, we evaluated the robustness of the model to single gain- and loss-of-function mutations, as well as its robustness to changes in the components of the logical update rule. Besides the study of these properties of the system, the model was compared with the expected effect of the extracellular microenvironments, gain- and loss-of-function mutations, and mechanical forces associated to several diseases in humans.

Regarding the validation of our model, the standard way of doing it is by comparing the specific effects of gain and loss-of-function mutations as reported in the references with their simulated effect. Furthermore, we also simulated the conditions that have been associated with several diseases related o EndMT and compared the simulated dynamic behavior of our model with the clinical observations of the pathologies.

### Formalization of the Molecular Regulatory Network as a Discrete Dynamical System

By assuming that every molecule in a regulatory network has a concentration threshold that must be exceeded in order to have an effect, it makes sense to use the formalism of a BN, where each molecule is represented by a node that can be either active or inactive, represented by 1 or 0, respectively. Let B = { 0,1 } and ${\mathbb{Z}}_{\leq n}^{+}=\left\{1,2,\ldots ,n\right\}$ a set of labels. A *state* of a BN is an n-tuple *x* = (*x*_1_,*x*_2_,…,*x_n_*) such that *x* ∈ B^*n*^, and each component *x_i_* of a state *x*, represents *the activation state of variable i*. To relate a synchronous BN with a molecular network, we interpret that variable *i* denotes a molecule included in the network. A BN is then a set of functions that contains for each variable *i* in the network an update rule *f*_*i*_:B^*k*^→B where *k* is the number of nodes that regulate variable *i*, and the n-tuple is an ordered list of the states of the nodes that regulate node *i*. The dependency of the state of activation of each node on the discrete time parameter *t* is denoted as *x_i_* (*t*), and obeys the update rule given by *f_i_*, such that for all *t* ∈ Z:

$$x_{i}\left(t+1\right)=f{\left(x\left(t\right)\right)}_{i}=f_{i}\left(r_{i}\left(t\right)\right).$$

When no race conditions or important cyclic behaviors are expected from the simulated dynamic behavior of the model, it is convenient to update all nodes simultaneously obtaining a deterministic discrete dynamic system. A *synchronous* BN with *n* components is a function *f*:B^*n*^→B^*n*^, where the *i-th component of f* is a function *f_i_* such that *f_i_*(*x*) = *f*(*x*)*_i_*. That is for all *t* ∈ *ℤ*

$$x\left(t+1\right)=f\left(x\left(t\right)\right)=\left(f_{1}\left(r_{1}\left(t\right)\right),f_{2}\left(r_{2}\left(t\right)\right),\ldots ,f_{n}\left(r_{n}\left(t\right)\right)\right).$$

BNs encode regulatory interactions among the molecules that compose them. Node *j activates* node *i* if there exists a pair of network states *x*, *y* that differ only in the state of activation of variable *j*, where *x_j_* = 0 and *y_j_* = 1, such that *f_i_*(*y*) – *f_i_*(*x*) > 0. Conversely, node *j inhibits* node *i* if there exists a pair of network states *x*, *y* that differ only in the state of activation of variable *j*. Specifically, *x_j_* = 0 and *y_j_* = 1, such that *f_i_*(*y*) – *f_i_*(*x*) < 0. Node *i* both activates and inhibits node *j* if there exists a pair of network states *x*, *y* that differ only in the state of activation of variable *j*. Specifically, *x_j_* = 0 and *y_j_* = 1, such that *f_i_*(*y*) – *f_i_*(*x*) > 0, and there exists another pair of network states *p*. *q* that differ only in the state of activation of variable *j*. Specifically, *p_j_* = 0 and *p_j_* = 1, such that *f_i_*(*q*) – *f_i_*(*p*) > 0. An *interaction* denoted as the pair (*i*, *j*), *i*,*j* ∈ *ℕ*_≤*n*_ is *functional* if variable *j* activates or inhibits variable *i*, or both.

BN models as defined above are deterministic and finite systems, thus simulating the dynamic behavior from any given initial state of the network leads to an attractor. A *fixed point attractor* is a state *s*∈B^*n*^ such that *f* (*s*) = *s*. If we define *f ^ol^* as the *l*-th iterate of the function *f* such that *f ^ol^* = *f* (*f ^o^*^(^*^l^* ^–^^1)^). Then, an *attractor* is a set of states *A*⊆B^*n*^, such that *f ^ol^*(*x*) = *x* for any state *x* ∈ *A*. Furthermore, *l* is the size of the attractor and for any $j\in {\mathbb{N}}_{<l}^{+}$, *f ^oj^*(*x*)∈*A*.

It is a standard practice to interpret fixed point attractors as the stationary patterns of molecular activation observed in a given regulatory network, and attractors of larger order as cyclic patterns of molecular activation (Álvarez-Buylla Roces et al., 2018; Yang and Albert, 2019). In the present study, we were able to assign to all attractors a biological interpretation in term of either a cell type or a cellular behavior.

We defined each component *f_i_* of the update rule *f* as follows: In the simplest case, the node *N*1 is only regulated by *R*1, then *f_N_*_1_ = *x_N_*_1_ (*t* + 1) = *x_R_*_1_(*t*). However, when the number of regulators is greater than one, we find groups of active and inactive regulators that are sufficient to activate a given node. We then represent such group as a logical expression where if all the regulators of the group are active or inactive, as needed, then the node is active. For instance, if *N*2 is regulated by the activators *A*1, *A*2, and *A*3 that form a complex, and the formation of such complex is inhibited by *I*1, then *f*_*N*2_ = *x*_*N*2_(*t*+1) = *x*_*A*1_(*t*) ∧ *x*_*A*2_(*t*) ∧ *x*_*A*3_(*t*) ∧ ¬*x*_*I*1_(*t*). If there are several groups of molecules that are sufficient to activate the node, then those groups form an OR expression. For example, if *N*3 represents a gene that can be activated either by *A*4 if *I*2 is absent, or independently by *A*5, then *f*_*N*3_ = *x*_*N*3_(*t*+1) = (*x*_*A*4_(*t*) ∧ ¬*x*_*I*2_(*t*)) ∨ *x*_*A*5_(*t*). Additionally, some nodes are regulated at transcriptional, posttranslational and protein levels and can be formalized using an AND expression. For example, if the transcription of node *N*4 is regulated by *TF*1 or *TF*2, its splicing is regulated by *SF*1, and also *MPK*1 activates the protein by phosphorylation and *PF*1 causes its proteolysis. Then *f*_*N*4_=*x*_*N*4_(*t*+1)=(*x*_*TF*1_(*t*)∨*x*_*TF*2_(*t*))∧*x*_*SF*1_(*t*)∧(*x*_*MPK*1_(*t*)∧¬*x*_*PF*1_(*t*)).

The molecular basis of our regulatory network is sufficient to specify the direction and sign for most of the interactions, as well as to specify most of the components of the logical update rule of the model. Nevertheless, in some cases the published information was not sufficient to unequivocally determine the sign of an interaction or an update rule. In these cases, we adjusted the system by assuming that the dynamic behavior of our model must reach fixed or cyclic patterns of molecular activation that correspond to the expected cell marker expression for Phalanx, Stalk, and Tip EC behaviors, as well as mesenchymal cells.

For the interested reader, the BoolNet, and GINsim versions of the discrete model are available for download at https://github.com/NathanWeinstein/EndMT.

### Molecular Pattern Identification

We labeled the attractors according to the molecular activation patterns associated to specific cell types or cell states. Notably, these labels are not mutually excluding; a given network state may fit more than one label. In the following paragraphs, we describe the possible labels that might be assigned to network states. Furthermore, some of the attractors are cyclic in nature, therefore, we applied a label to a cyclic attractor only if it was possible to apply the label to each one of the states that composes the cyclic attractor.

It is known that all ECs express VE-cadherin, PECAM1, TIE2, and VEGFR2. These molecules, in turn, are activated by the combined presence of the transcription factors GATA2, and FLI1. Hence, whenever a network state has these two nodes in an active state, we say that such network represents an EC. Some mesenchymal cells express GATA2 and FLI1, but they also express fibroblast specific protein-1 (FSP-1), αsmooth muscle actin (αSMA), Smooth muscle-22α (SM22α), encoded by transgelin (TAGLN), and fibronectin (Kamata et al., 2014). The precise mechanism by which mesenchymal markers are expressed during EndMT has not been fully elucidated. However, SNAI1, SNAI2, TWIST1, ZEB1, and ZEB2, which are also expressed by certain ECs, have been used experimentally as mesenchymal markers (Magenta et al., 2011; Welch-Reardon et al., 2014; Mahmoud et al., 2016). Because of these, we identify as a mesenchymal cell all those network states where ZEB1, ZEB2, TWIST1, and either SNAI1 or SNAI2 are active. Phalanx ECs are the quiescent and tightly-bound ECs that form a layer that functions as a barrier. We identify as Phalanx ECs those states where there is an absence of NPR1, CTNNB, SNAI1, SNAI2, while GATA2 and FLI1 are present. The absence of the first set of markers is important because SNAI1 and SNAI2 inhibit the transcription of VE-cadherin (Lopez et al., 2009; Cheng et al., 2013), and other important components of endothelial adherence and tight junctions (Laakkonen et al., 2017). Also, CTNNB activates the transcription of SNAI2 and TWIST1, while CTNNB and LEF1 induce EC proliferation by activating the transcription of *Cyclin D1*. Finally, NRP1 is a marker for Tip EC behavior (Aspalter et al., 2015), (Phng et al., 2009). Stalk cells are activated ECs that trail ECs. These cells express FLI1, GATA2, and JAG1, yet they do not express NPR1 (del Toro et al., 2010; Blancas et al., 2012). Finally, Tip cells are activated ECs that grow filipodia. Here, we use the presence of FLI1, GATA2, NRP1, and ETS1 to identify Tip cells. NRP1 is a recognized Tip cell marker (del Toro et al., 2010; Blancas et al., 2012) and Tip cells must express DLL4, which requires ETS1 activity (Wythe et al., 2013).

The *basin of attraction* of an attractor is the group of states that converges to that attractor. These states include the attractor itself. In models where an attractor corresponds to just one cell type (see for example Weinstein et al. (2015)), it is customary to characterize the basins of attraction. In the present model, however, a given attractor may correspond to more than one label, and *vice versa*, one label can be assigned to more than one attractor. Henceforth, it is necessary to define a *trap space* of any given cell type or behavior *c*. This trap space is the union of the basins of attraction of the fixed and cyclic behaviors that can be classified as *c*. We estimated the size of each trap space by first generating 10^7^ random network states. For each state, we simulated the behavior of our model until reaching an attractor. We then classified the attractor and calculated the fraction of the sampling space covered by each trap space.

### Robustness of the Model

Evolution has made biological organisms resilient to several perturbations such as mutations and fluctuations in the concentration or level of molecular activation, while at the same time remaining sensitive to changes in the concentration of key molecular signals used to regulate its development. We refer to this property as selective robustness. Specifically, the systems affected by EndMT resist most changes in the extracellular microenvironment, single gain and loss-of-function mutations, as well as parameter variation. Substantial alterations occur only when a critical molecule or interaction is affected, or when several molecules are affected simultaneously. Therefore, the molecular mechanisms involved in EndMT regulation exhibit selective robustness. For clarity, we need to specify the trait we test for robustness, as well as the nature of the perturbations we use to assess such robustness. Moreover, it is also necessary to define a method to quantify robustness (Félix and Barkoulas, 2015). Hence, we measured the robustness of the network in the following ways:

1. The robustness of the cell types, as measured by the percentage of gain- or loss-of-function mutations the system is able to resist without the loss of a specific stationary or cyclic pattern of molecular activation.
2. The robustness of the cell types to random changes in the update rule. This was done by generating a population of 100,000 instances of the models, but each instance affected by a single bit-flip in a random component of the update rule. The mean number of attractors for all the networks in the population were calculated. We say in this case that a cell type is robust if the mean of the population is closer to the nonperturbed model, and also if the variance is small.
3. The sensitivity of each component of the update rule to molecular activation noise. For each update rule component, namely each *f_i_* ∈ *f*, we generated 500,000 random initial states, and for each one of those initial states *s*, a variant *s'* is generated with a one bit flip. Then, we applied the update rule to both *s* and *s'* and calculated the sensitivity of *f_i_* as the fraction of initial states where *f* (*s*)*_i_* ≠ *f* (*s'*)*_i_*. Additionally, we calculated the sensitivity of each update rule component when flipping from 2 to 15 bits of *s* to obtain *s'* in order to observe how the sensitivity of each update rule is affected by different levels of molecular activation noise. For each component and each number of flipped bits we used 20,000 random initial states.
4. The robustness of each cell type in response to perturbations in the molecules that represent the extracellular microenvironment and the main transcription factors involved in maintaining EC identity. Such nodes are *DLL4, FGF2, FLI1, GATA2, HIF1α, PDGF*_*AB, TGFβ, VEGFA, WNT5b*, and *WNT7a*. For each of the patterns classified as a cell type or cellular state, we tested all possible combinations of perturbations in the aforementioned nodes and let the system converge. Here, the robustness is the fraction of the perturbations that were absorbed by the system, such that the network reached the original cell type or behavior before the perturbation.

### Libraries for the Dynamical Analysis

We used *GINsim* (Naldi et al., 2009) to find and analyze the feedback circuits of our model. Then, we used the R package *BoolNet* (Müssel et al., 2010) to find the attractors using a heuristic method that formulates the attractor search as a boolean satisfiability (SAT) problem that is solved using the PicoSAT solver (Biere, 2008; Dubrova and Teslenko, 2011). We also used *BoolNet* to simulate mutations and perturbations. The analysis of the perturbations that cause changes in cell type and behavior required preparing a function for parallel processing, and for this we used the R package *doParallel* (Weston and Calaway, 2019). We also used the R package *ggplot2* (Wickham, 2011) to create graphics. Lastly, we used the R package *xtable* (Swinton, 2014) to export matrices and data frames from R into LaTeX. The scripts and the data generated by the scripts are freely available at: https://github.com/NathanWeinstein/EndMT.

## Results

### Molecular Basis of the Regulatory Network

EndMT is defined by the loss of EC adhesion, the conversion of endothelial apical-basal polarity to front end-back end polarity, and a marked decrease in EC markers accompanied by increased MC marker expression. During EndMT, the signaling pathways of TGF, WNT, NOTCH, VEGF, FGF, TNF, and PDGF modulate the activity of the transcription factors FLI1 and GATA2 that are essential for EC identity, as well as the activity of SNAI1, SNAI2, TWIST1, ZEB1, ZEB2, and LEF1 that are necessary for mesenchymal cell differentiation. Importantly, these transcription factors form a complex regulatory network that we have uncovered here. The following sections include the mechanism by which these and other relevant molecules regulate each other.

#### EC Adhesion

In stable and mature blood vessels, ECs are interconnected, forming a barrier that separates blood or lymph from the surrounding tissue. Additionally, ECs are covered by a basement membrane, and at least partially covered by mural cells. Many of the proteins that compose the transmembrane complexes that bind ECs together are expressed only in ECs, and are thus used as EC markers. Both EndMT and EC activation reduce EC adhesion and increase the EC barrier permeability; however, only EndMT causes ECs to completely detach from the endothelial monolayer.

EndMT represses the expression of many of the proteins that compose intraendothelial junctions resulting in loss of EC adhesion and identity. ECs are connected by junctional proteins, which assemble to form adherens junctions (AJs) that link the cytoskeletons of adjacent ECs; by tight junctions (TJs) that function as a selectively permeable barrier between ECs; and by gap junctions (GJs) that function as selective ion channels (Radeva and Waschke, 2018). Furthermore, focal adhesions (FAs) anchor ECs to the basement membrane, but they can also be located between ECs where they function as important regulators of the microvascular function (Wu, 2005).

Vascular endothelial cadherin (VE-cadherin) is one of the main components of endothelial AJs (Giannotta et al., 2013). *α,β* and γ-catenins, α-actinin and vinculin anchor VE-cadherin to actin. VE-cadherin can also recruit the desmosomal proteins desmoplakin and vimentin. Intermediate filaments composed of vimentin may be linked to endothelial AJs *via* plakoglobin/desmoplakin or p0071 forming junctional structures called *complexus adherens* (Wallez and Huber, 2008). Moreover, VE-PTP inhibits VEGFA-mediated phosphorylation of VE-cadherin, thus stabilyzing endothelial AJs (Bazzoni and Dejana, 2004). Furthermore, VE-cadherin, PECAM1, and VEGFR2 form a junctional mechanosensory complex (Conway et al., 2013; Kutys and Chen, 2016). Nectins are one of the main components of AJs, are linked to actin microfilaments through Afadin, and also form interendothelial bonds.

Tight junctions also include proteins that form bonds at the interendotelial cleft, forming a physical barrier that prevents solutes and water from freely crossing the EC sheet. The number of TJs at an interendotelial cleft is proportional to the shear stress applied to the endothelial sheet by blood flow. The proteins that compose TJs include Claudins, Ocludin, JAMS, ESAM, and Nectins. Those proteins are linked to numerous intracellular partners, including AF-6/afadin, cingulin, the antigen 7H6, PAR-3, ZO-1, ZO-2, and ZO-3, forming a molecular complex (Wallez and Huber, 2008). The barrier forming Claudins CLDN3, CLDN5, and CLDN11 are expressed by ECs. Occludin (OCLN) increases TJ barrier function and is one of the main molecules involved in the regulation of endothelial layer permeability. The expression of ocludin is upregulated by Angiopoietin 1 (ANGPT1), and further stabilized by angiotensin-2 (AT2) binding to type 1 angiotensin receptor (ATR). VEGFA downregulates OCLN by inducing OCLN proteolysis through activation of the urokinase (uPA)/uPAR system and also by PKC-mediated phosphorylation. OCLN is also regulated by monocyte chemoattractant protein-1 (MCP-1/CCL-2), histamine, oxidized phospholipids, lysophosphatidic acid, and shear-stress (González-Mariscal et al., 2008; Wallez and Huber, 2008; Radeva and Waschke, 2018). The junctional adhesion proteins F11R (JAM-A), JAM2 (VE-JAM or JAM-B), JAM3 (JAM-C), and the related protein ESAM (EC adhesion protein) from the immunoglobulin superfamily are important components of endothelial TJs that regulate paraendothelial permeability, leukocyte trafficking and TJ dynamics (Wallez and Huber, 2008; Rahimi, 2017).

FAs are composed of α and β integrin heterodimers that bind several ECM components, as well as TJ components and several intracellular proteins. Those adhesive integrin interactions contribute to the maintenance of endothelial barrier function, and the loss of integrin-matrix adhesion results in leaky microvessels (Wu, 2005; Izawa et al., 2018). ECs express multiple integrins that assemble into several different heterodimers. The extracellular domains of many integrins have a high binding affinity for the Arg-Gly-Asp (RGD) sequence and are able to interact with several matrix proteins. However, certain heterodimers exhibit a higher affinity for a specific protein including *α*6*β*1 and *α*6*β*4 that favor laminin, *α*1*β*1 and *α*1*β*2 that tend to bind collagen, *αvβ*3 and *αvβ*5 that exhibit affinity to vitronectin, as well as *α*3*β*1 and *α*5*β*1 that favor fibronectin (Wu, 2005). Focal adhesion kinase (FAK) is another important FA component. The N-terminal domain of FAK contains a region called FERM homology that exhibits a high binding affinity for growth factor receptors and integrins. The C-terminal domain contains a noncatalytic region, also referred to as FRNK (FAK-related nonkinase), that carries a FAT sequence that directs FAK to adhesion complexes and provides docking sites for other cytoplasmic proteins. FAK activation, triggered by phosphoryation regulates endothelial barrier function either increasing or decreasing permeability depending on the site of phosphorylation and the context. When VEGFA binds VEGFR2, it causes a conformation change that exposes an integrin α*vβ*3 binding site. Integrin α*vβ*3 then binds VEGFR2, recruits FAK and promotes the activation of several signaling pathways that lead to increased microvascular permeability. VEGFA also causes phosphorylation-coupled FAK activation and relocation from the cytoplasm to focal contacts.

#### EC Polarization

Certain cellular processes including asymmetric cell division, cell migration, and barrier formation require the asymmetric organization of components within a cell. In stable blood vessels, ECs have an apical (luminal) membrane domain, an interendothelial (lateral) membrane domain, and a basal membrane domain. This organization results in a luminobasal or apicobasal EC polarity. During angiogenesis, the cytoskeleton of tip cells and stalk cells undergoes several changes that result in transient front-to-rear EC polarity which is necessary for collective directed migration (Ebnet et al., 2018). Many of the molecules involved in EC polarization are implicated in lumen formation and also regulate endothelial permeability linking these processes (Lizama and Zovein, 2013).

Both angiogenesis and vasculogenis involve cord hollowing, a process that results in lumen formation. Prior to lumen formation, the ECs that compose the segment must acquire an apicobasal polarity (Lizama and Zovein, 2013; Ebnet et al., 2018). The molecular signaling pathways involved in EC polarization and lumen formation are largely unknown and are subject to current research (Norden et al., 2016; Szymborska and Gerhardt, 2018). During early embryonic vasculogenesis, *β*1 integrin (ITGB1), RAS interacting protein 1 (RASIP1), and partitioning defective 3 (PAR3) interact to establish EC apicobasal polarity before epithelial lumen formation (Herbert and Stainier, 2011). VE-cadherin acts as a positional cue to attract and organize the proteins involved in EC polarization. Accordingly, loss of VE-cadherin function prevents apicobasal EC polarization and EC agglomerations from developing a vascular lumen. VE-cadhein directly interacts with many proteins involved in EC polarization such as PAR3, PARD6A (PAR6), MPP5 (PALS1), and KRIT1 (CCM1) (Giannotta et al., 2013; Lizama and Zovein, 2013; Brinkmann et al., 2016). VE-cadherin recruits the sialomucins CD34 and PODXL (Podocalyxin) to EC-cell contact sites. Sialomucins contain negative charges that cause repulsive forces and initiate adjacent EC membrane separation. Later, VE-cadherin is involved in Moesin, F-actin and nonmuscle myosin II recruitment to induce lumen expansion and stabilization. Other proteins involved in lumen expansion and stabilization include Protein kinase C (PKC) that links CD34 to the actin cytoskeleton through Moesin phosphorylation, and ROCK, that is also necessary for nonmuscle myosin II recruitment (Lizama and Zovein, 2013).

During the initial stages of angiogenesis, tip cells form filopodia and lamellipodia and orient them following the gradient of a vascular growth factor, typically VEGFA. The Ras homologue gene (Rho) and Ras-related protein (Rap) families of small G proteins are important mediators of VEGFA signaling in ECs (Shimizu et al., 2018). The Rho GTPases RhoA, Rac1, and Cdc42 interact with integrins at FAs where actin accumulates to initiate the formation of filopodia and lamellipodia (Lizama and Zovein, 2013). Tip cells then migrate toward the source of the morphogen trailing stalk cells. During sprout elongation, the elastic properties of the cytoskeleton of the ECs that conform the sprout have to be tightly regulated (Szymborska and Gerhardt, 2018). *In vitro*, EC sprout elongation requires a reduction of EC contractility mediated by the downregulation of Rho kinase (ROCK) and myosin light chain 2 (MLC2). Another important molecular mechanism that increases EC contractility involves RAP1, which induces the formation of a RAF1-VE-cadherin complex that recruits ROCK (Szymborska and Gerhardt, 2018). KRIT1 is an effector of RAP1, which upon activation interacts with *β*-catenin and afadin. Additionally, KRIT1 stabilizes endothelial junctions by recruiting RAP1 that stabilizes and concentrates VE-cadherin. KRIT1 also recruits CCM2 to the junction where it inhibits RHOA to further stabilize the junction. Another important function of KRIT1 is to prevent the activation of the canonical WNT signaling pathway by sequestering β-catenin (Wilson and Ye, 2014).

#### Key Transcription Factors for Endothelial and Mesenchymal Identities

The specification and maintenance of EC identity requires the function of ETV2, FLI1, ERG, ETS1, and other members of the E26 transforming specific (ETS) family of transcription factors; all of them share a core GGAA/T DNA-binding motif (Craig and Sumanas, 2016). ETV2 function is required for endothelial specification during early embryonic development in both mice and zebrafish (Abedin et al., 2014), and it is also necessary for vascular regeneration after an injury (Park et al., 2016). ETV2 directly binds to the promoters of *Cdh5* (VE-cadherin), *Tie2*, *Kdr*(VEGFR2), *Scl, Gata2, Meis1, Dll4, Notch1, Nrp1/2, Flt4, RhoJ, Mapk*, and *Fli1* (Oh et al., 2015). Later, during embryonic development, ETV2 is no longer expressed and FLI1 maintains endothelial identity by binding to the promoters of *Cdh5, Tie2, Cd31*(PECAM1), *Erg* and *Fli1*, activating their expression as well as its own (Abedin et al., 2014). Notably, diminishing the expression of FLI1 and ERG triggers the EndMT (Nagai et al., 2018).

ETS1 exhibits functional redundancy with ETS2, is expressed during angiogenesis, and is involved in the regulati6on of EC survival, migration, and proliferation. ETS1 induces the expression of several matrix metalloproteinases (MMPs), integrins, and *NRP1* (Teruyama et al., 2001; Craig and Sumanas, 2016). Then, GATA2 belongs to the C2H2 zinc-finger class of transcription factors and is also involved in the regulation of EC identity. Importantly, the loss of GATA2 in ECs triggers EndMT. In ECs, GATA2 activates the transcription of *Emcn* (Endomucin, interferes with FJ assembly), *Cdh5, Pecam1, Vegfr2, Nrp1, vWF*, and *Gata2* itself (Kanki et al., 2011; Coma et al., 2013). It is also important to mention that GATA2 and FLI1 activate the transcription of each other (Pimanda et al., 2007b).

Five transcription factors have been associated with EndMT. Four of them, SNAI1 (SNAIL), SNAI2 (SLUG), ZEB1, and ZEB2 (SIP1), contain four to six E2â€ box DNA binding zinc fingers, and a SNAG domain involved in transcriptional repression. The other transcription factor is the basic helix-loop-helix (bHLH) TWIST1 (Gong et al., 2017; Jackson et al., 2017; Sánchez-Duffhues et al., 2018). SNAI1, SNAI2, and TWIST directly repress the transcription of VE-cadherin (Lopez et al., 2009; Cheng et al., 2013). Other components of endothelial AJs and TJs are also downregulated during EndMT. However, in most cases, the molecular mechanism has not been fully elucidated. For instance, CLDN5 is downregulated by SNAI1 (Kokudo et al., 2008) and SNAI2 (Laakkonen et al., 2017), yet it is well recognized that VE-cadherin is a key component of endothelial junctions that integrates molecular and mechanical signals. VE cadherin is involved in EC identity, quiescence, migration and polarization. Therefore, loss of VE-cadherin function explains several of the cellular processes involved during EndMT.

Both SNAI1 and SNAI2 proteins bind to E2 boxes in promoters that regulate *Snai1* and *Snai2* expression (Chen and Gridley, 2013b). SNAI1 and SNAI2 directly suppress each other's transcription during chondrogenesis (Chen and Gridley, 2013b; Chen and Gridley, 2013a). SNAI1 (Peiro et al., 2006) and TWIST1 (Yu et al., 2013; Forghanifard et al., 2017) directly repress the transcription of *Snai1*. However, E47 binds TWIST1 forming a dimer that binds to the *Snai1* promoter and activates its expression (Yu et al., 2013). In certain tumor cells, SNAI1 upregulates ZEB1 and ZEB2 expression (Guaita et al., 2002; Takkunen et al., 2006). In contrast, in melanoma cell lines, SNAI1 does not activate the transcription of ZEB1 (Wels et al., 2011), thus, we have not included this interaction in our model. SNAI2 (Kumar et al., 2015) and TWIST1 (Casas et al., 2011) directly activate the transcription of *Snai2*. SNAI2 also directly induces the transcription of ZEB1 (Wels et al., 2011). The molecular mechanism that causes loss of FLI1, ERG, and GATA2 expression to induce EndMT remains obscure. Nonetheless, it is known that GATA2 siRNA leads to increased SNAI1 and SNAI2 expression, and GATA2 binds to the proximal promoter of SNAI2 (Kanki et al., 2011). Additional interactions have been reported for other cell types. In hematopietic stem cells, for example, TWIST1 binds to the promoter of *Gata2* and induces its transcription (Kulkeaw et al., 2017), while in nasopharyngeal carcinoma cells, GATA2 induces EMT by binding to the promoter of *Twist1* and activating its expression (Wang et al., 2017b). Furthermore, ETS1 and ZEB2 activate each other's transcription (Katoh and Katoh, 2009; Yalim-Camci et al., 2019).

#### The Molecular Signaling Pathways Involved in EndMT Regulation

In a previous model of endothelial behavior during angiogenesis (Weinstein et al., 2017), the TGF, NOTCH, WNT, VEGF, FGF, and HIF signaling pathways were described in detail. Thus, we will focus now on their roles during the EndMT.

The TGF signaling pathway is of central importance for the regulation of EC plasticity and EndMT (Dejana et al., 2017). When a TGF or a BMP ligand binds to a TGF receptor complex, it causes the activation of several signaling pathways that mediate TGF-induced EndMT, among them SMAD, MEK, p38 MAPK, and PI3K signaling (Medici et al., 2011). Some of the key components of TGF signaling involved in the regulation of EndMT include the ligand TGF*β*2 (Chen et al., 2012), type I receptors ALK1 and ALK5 (TGFBR1), the type II receptor TGF*β*R2, as well as SMAD2, SMAD3, and SMAD4 (Medici et al., 2011; Chen et al., 2012). SMAD2 and SMAD3 activate the transcription of SNAI2, while SMAD4—which is a co-SMAD that allows other SMADs to activate the transcription of target genes—is required for TGF-induced SNAI1 expression (Cooley et al., 2014). The expression of ZEB2 is induced by TGF signaling and its promoter contains SMAD binding sites (Katoh and Katoh, 2009). Furthermore, ZEB1 and ZEB2 bind SMADs forming transcriptional regulation complexes (Grabitz and Duncan, 2012). Also, TGF*β*2 also induces inhibitory VEGFA splicing (Weinstein et al., 2017).

FGF signaling modulates EC and PC function and behavior. When an FGF ligand, like FGF2, binds to an FGF receptor such as FGFR2, it causes FRS2-mediated ERK and PI3K signaling pathway activation (Yang et al., 2015). FGF signaling inhibits EndMT by downregulating TGF signaling; FGF2 activates the transcription of miRNAs from the *let7* family, especially *let7b* and *let7c*, which prevents the expression of TGFBR1 (Chen et al., 2012). FGF2 also increases the expression of *mir-20a*, another miRNA that prevents the expression of TGFBR1, TGFBR2 and SARA (Smad anchor for receptor activation) (Correia et al., 2016). In addition to RNA silencing, FGF2 activates the Ras-MAPK signaling pathway that regulates TGFB1-induced SMAD2 phosphorylation in lymphatic ECs (Ichise et al., 2014). Another important function of FGF signaling in ECs is to activate the transcription of VEGFR2. FGF activates ERK signaling, which then activates several transcription factors from the ETS family including ETS1 and ETV2 that activate *Vegfr2* transcription (Murakami et al., 2011; Yang et al., 2015).

Insufficient oxygen availability (hypoxia) in the cells that compose the tissue surrounding a network of capillaries triggers angiogenesis. HIF1, composed of subunits HIF1*α* and HIF1*β*, is a key mediator of the cellular response to hypoxia. Hypoxia prevents the PHD-mediated proteasomal degradation of HIF1*α*, a molecule that directly activates the transcription of *VegfA* (Forsythe et al., 1996; Kumar et al., 2014). When ECs themselves are exposed to hypoxia, it may cause senescence, increased apoptosis and necrosis rates due to augmented oxidative stress and irreparable DNA damage, or angiogenesis and proliferation, depending on the duration and severity of the reduction in oxygen availability (Baldea et al., 2018). Under certain circumstances, hypoxia causes EndMT. In this case, HIF1*α* directly binds to the promoter region of *Snai1* and induces its transcription (Xu et al., 2015). Hypoxia also induces the expression of SNAI2 and TWIST1 in ECs (Xu et al., 2015). Additionally, during EMT (Yang and Wu, 2008) and also during EndMT associated with pulmonary arterial hypertension, HIF*α* directly induces the expression of TWIST1 (Zhang et al., 2018). Furthermore, the proximal promoter region of ZEB2 contains a HIF1*α*-binding site (Katoh and Katoh, 2009). Finally, HIF1 is an important inducer of EC differentiation since HIF1*α* binds to the *Etv2* promoter and activates its transcription. ETV2, in turn, activates the transcription of *Fli1* (Oh et al., 2015).

VEGF signaling is involved in EC activation during vascular remodeling. Typically, during angiogenesis VEGFA binds to a VEGFR2 homodimer and activates PLCγ, and TSAd-AKT signaling (Simons et al., 2016). VEGFA signaling strengthens the EC identity by activating the expression of GATA2 (Coma et al., 2013). Further, VEGFA-VEGFR2 signaling phosphorylates and activates STAT3 (Chen et al., 2008), which then activates the transcription of SNAI1 in HeLa cells (Saitoh et al., 2016). Additionally, the VEGF co-receptor NRP1 is a key molecule that promotes tip cell behavior and inhibits stalk cell behavior by limiting SMAD2/3 phosphorylation (Aspalter et al., 2015). However, NRP1 also acts as a co-receptor for TGF*β*1 and is necessary for TGF*β*-mediated EndMT (Matkar et al., 2016).

Notch signaling is required for EndMT by the cardiac cushions during early cardiac valve development. The signaling of this pathway is initiated when a ligand (DLL4) binds to a Notch receptor (NOTCH1). Then, the receptor is cleaved into an intracellular domain, a transmemrane domain, and an extracellular domain. NOTCH1 activation leads to increased SNAI2 expression (Niessen et al., 2008), as well as increased SNAI1 stability and nuclear retention. The intracellular domain of NOTCH1 forms a complex with *β*-catenin and TCF4 that activates the transcription of AKT2. This molecule then inhibits glycogen synthase kinase 3 (GSK3*β*)-mediated proteolysis and translocation of SNAI1 from the nucleus to the cytoplasm (Frías et al., 2016). Furthermore, Notch signaling induces the transcription of both subunits of the nitric oxide (NO) receptor soluble guanylyl cyclase (sGC), namely GUCY1A3 and GUCY1B3. Also, this signaling induces Activin A, consequently promoting both NO production and the transcription of its receptor, which are necessary for EndMT to occur in the developing AVC (Chang et al., 2011). In response to an increase in shear stress, NOTCH1 activation leads to the formation of GTPase signaling complexes at AJs composed of the NOTCH1 transmembrane domain, VE-cadherin, the guanine nucleotide exchange factor Trio, and the tyrosine phosphatase LAR that activates RAC1 to stabilize adherens junctions (Fischer and Braga, 2018). NOTCH also induces the transcription of *Vegfr1*. VEGFR1 inhibits VEGFA-VEGFR2 signaling by reducing the amount of VEGFA available to bind VEGFR2 (Funahashi et al., 2010). The Notch-regulated ankyrin repeat protein (NRARP) links NOTCH and WNT signaling. Dll4-NOTCH1 signaling induces *Nrarp* expression in ECs. NRARP negatively regulates Notch signaling by destabilizing the Notch intracellular domain and positively regulates Wnt signaling by increasing the stability of the LEF1 protein (Ishitani et al., 2005; Phng et al., 2009). Finally, another important function of NOTCH signaling in stalk cells is to negatively regulate the expression of NRP1 (Aspalter et al., 2015).

Canonical Wnt signaling is initiated by a WNT ligand, which is usually WNT7A or WNT3, and leads to the stabilization of CTNNB (*β*-catenin). Like Notch signaling, canonical Wnt signaling also causes GSK3*β* phosphorylation, allowing the accumulation and nuclear localization of SNAI1 and SNAI2 (Wu et al., 2012; Menezes, 2014). Further, the complex formed by CTNNB and TCF activates the transcription of many of the genes regulated by canonical Wnt signaling (Menezes, 2014), including SNAI2 (Conacci-Sorrell et al., 2003), TWIST1 (Howe et al., 2003), and ZEB1 (Sánchez-Tilló et al., 2011; Sanchez-Tillo et al., 2014). CTNNB and TCF also induce the transcription and activation of LEF1. During EMT, LEF1 activates the transcription of *Snai2* and *Zeb1* even in the absence of both *β* and γ-catenins (Kobayashi and Ozawa, 2018). Other WNT ligands including WNT5a, WNT5b, and WNT11 activate the noncanonical planar cell polarity (PCP) and *CA*^+2^ WNT signaling pathways that also activate the Activator protein 1 (AP-1) transcription factor (Nishita et al., 2010). AP-1 binding sites exist in the promoter regions of *Snai1* and *Snai2*, and the inhibition of AP-1 results in reduced SNAI1 expression in mesenchymal cells (Nguyen et al., 2013). Moreover, WNT5b induces EndMT and SNAI1 expression in lymphatic ECs through the activation of WNT/*β*-catenin and PCP pathways. WNT5b also induces inhibitory VEGFA splicing through noncanonical WNT signaling (Weinstein et al., 2017).

The PDGF signaling pathway is involved in the regulation of pericyte recruitment during microvascular maturation, and EndMT-mediated pericyte differentiation from ECs (Gaengel et al., 2009; Chen et al., 2016). The signaling is initiated by a PDGF ligand that can be the PDGF-AB heterodimer, or one of four homodimers, namely PDGF-AA, -BB, -CC, and -DD. The tyrosine kinase receptors PDGFR*α* and PDGFR*β* dimerize after ligand biding. PDGF-AA forms PDGFR*αα*. PDGF-BB can form either PDGFR*αα*, PDGFR*ββ* or PDGFR*αβ* dimers. PDGF-CC forms PDGFR*αα*, or PDGFR*αβ* receptors. PDGF-DD signals specifically *via* the PDGFR*ββ* receptor, but is able to form the PDGFR*αβ* heterodimer. PDGF-AB forms PDGFR*αα*, or *PDGFRαβ* receptors. After activation and dimerization, PDGFRs can interact with signaling proteins that contain an SH2 domain, including FER, PI3K, PLC, SHP2, and SRC, leading to the activation of several signaling pathways, such as MAPK, PI3K-AKT-NF-*κ*B and PLCγ (Romashkova and Makarov, 1999; Papadopoulos and Lennartsson, 2018). ECs weakly express PDGFR*α* and PDGFR*β*. However, when brain ECs are exposed to PDGF-AB, it causes the activation of the transcription factor NF-*κ*B, which binds to the promoter of *Snai1* and activates its transcription, leading to EndMT (Liu et al., 2018). In spite of the fact that in human breast cancer cells, NF-*κ*B binds to the promoter regions of *Snai2, Twist1*, and *ZEB2* and activates their transcription (Pires et al., 2017), PDGF-AB does not increase the expression of *Snai2, Twist1*, and *ZEB2* in brain ECs (Liu et al., 2018). Additionally, NF-*κ*B directly activates the transcription of *Lef1* in chondrocytes (Yun et al., 2007).

During acute inflammation, TNF*α* and IL-1*β* cause NF-*κ*B-mediated EndMT by inducing the degradation of the inhibitory kB (Iκβα) protein, which sequesters NF-kB in the cytosol (Sánchez-Duffhues et al., 2018). Furthermore, inflammation may suffice to determine if an EC is activated or if it undergoes full EndMT. TNF*α* induces VE-cadherin internalization and degradation. Additionally, TNF*α* inhibits VE-cadherin expression by activating the transcription of hsa-miR-6086 (Cai et al., 2018). Shear stress and cyclic strain also modulate EndMT. Laminar shear stress activates the mechanosensitive transcription factors KLF2 and KLF4 that inhibit EndMT by downregulating AP1 and NF*κ*B. Also, KLF2 induces the expression of *Smad6, Smad7* and *VegfA*, which inhibit SMAD2 activity. Further, KLF4 activates the transcription of VE-cadherin, prevents the expression of genes regulated by SMADs by binding to the TGF*β* control element, and also impedes the transcription of mesenchymal genes by binding SMAD3. Cycle strain induces EndMT by Rho mediated VE-cadherin translocation from the membrane into the cytoplasm, causing the concentration of *β*-catenin in the nucleus to increase (Krenning et al., 2016). For simplicity, we only take into account one activating signal for AP-1, *β*-catenin, and NF-*κ*B.

### BN Model Assembly

**Figure 2:**
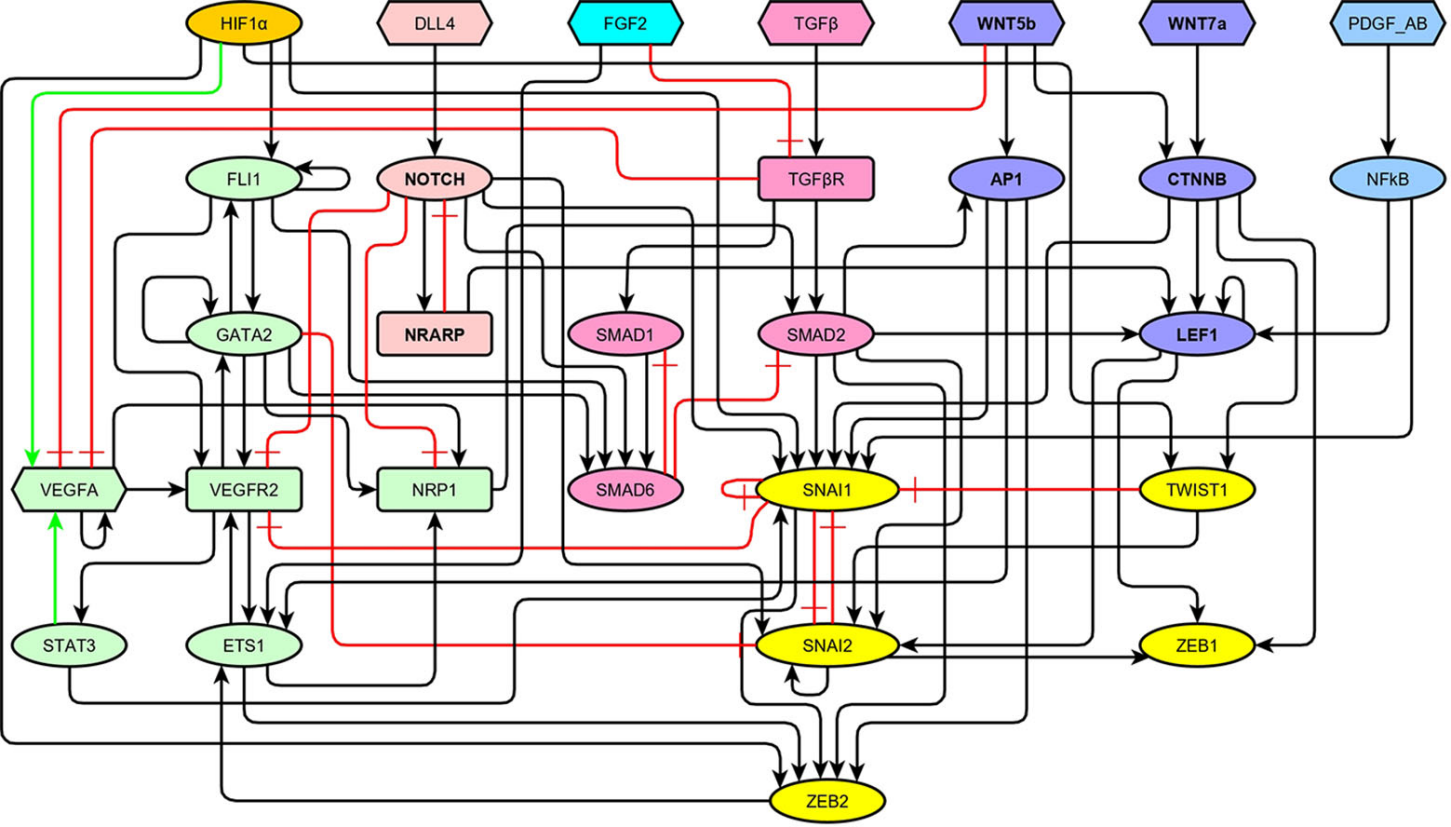
The topology of our model of the network of molecules involved in the regulation of endothelial-to-mesenchymal transition (EndMT) represented as a signed directed graph: Black arrows represent positive regulations, green arrows represent positive autocrine regulations, and red blunt arrows represent inhibitions. The VEGF signaling pathway and the main transcription factors involved in endothelial cell (EC) identity are shown in green, HIF1*α* is shown in orange, the NOTCH signaling pathway is shown in light red, FGF2 is shown in turquoise, the TGF signaling pathway is shown in pale magenta-pink, the WNT signaling pathway is shown in lavender, the PDGF signaling pathway is shown in light cyan-blue, and the main transcription factors involved in EndMT are shown in yellow.

As summarized in the previous section, a very large number of molecular components and pathways have been described to be involved in the regulation of EndMT and angiogenesis. In order to integrate their roles and understand their concerted action, we propose here a BN approach. For simplicity, we selected a subset of molecules. Specifically, we incorporated into our model only those molecules that are essential either due to their biological function, or due to their effect in the simulated dynamic behavior of our model. As a result, the regulatory network of EndMT includes 29 molecules connected by 77 regulatory interactions, as shown in **Figure 2**. The model encompasses molecules necessary for EC identity, the ligands that activate the VEGF, HIF, NOTCH, FGF, TGF, WNT, and PDGF signaling pathways, as well as the main transcription factors involved in EndMT. We did not include many EC and MC markers because they act as network sinks, and their activity can be inferred from that of the included transcription factors. Most of the 77 interactions represent direct transcriptional or posttranscriptional regulations. However, the interactions that connect ligands directly to transcription factors represent entire linear signaling pathways.

After the reconstruction of the regulatory network, we translated the information to construct a Boolean model, as described in Section 2.1. We used the molecular information outlined in Section 3.1 to obtain the logical rules. Additionally, the references we used to define each component of the update rule are specified in **Table 1**. However, in order for our model to reach fixed or cyclic patterns of molecular activation that correspond to the expected cell marker expression for Phalanx, Stalk and Tip EC behaviors as well as mesenchymal cells, we had to fix the rules in three instances (**Table 2**). As a result, the components of the update rule of the network are shown as follows in equations 1–29.

**Table 1:** References that serve as a base for each component of the update rule.

Molecule	References
AP1	Nishita et al. (2010); Sundqvist et al. (2013); Zhao et al. (2014)
CTNNB	Wu et al. (2012); Menezes (2014)
DLL4	Niessen et al. (2008); Chang et al. (2011)
ETS1	Murakami et al. (2011); Hollenhorst (2012); Chen et al. (2017); Yalim-Camci et al. (2019)
FGF2	Murakami et al. (2011); Chen et al. (2012); Ichise et al. (2014); Yang et al. (2015); Correia et al. (2016)
FLI1	Lelièvre et al. (2002); Pimanda et al. (2007b); Abedin et al. (2014); Oh et al. (2015); Tsang et al. (2017)
GATA2	Pimanda et al. (2007b); Kanki et al. (2011); Coma et al. (2013)
HIF1a	Kumar et al. (2014); Xu et al. (2015); Baldea et al. (2018)
LEF1	Ishitani et al. (2005); Medici et al. (2006); Yun et al. (2007); Phng et al. (2009)
NFκB	Liu et al. (2018)
NOTCH	Phng et al. (2009)
NRARP	Phng et al. (2009)
NRP1	Teruyama et al. (2001); Coma et al. (2013); Aspalter et al. (2015)
PDGF_AB	Liu et al. (2018)
SMAD1	van Meeteren and Ten Dijke (2012); Pardali et al. (2017)
SMAD2	van Meeteren and Ten Dijke (2012); Aspalter et al. (2015); Pardali et al. (2017)
SMAD6	Ishida et al. (2000); Pimanda et al. (2007a); Mouillesseaux et al. (2016)
SNAI1	Peiro et al. (2006); Julien et al. (2007); Kokudo et al. (2008); Medici et al. (2011); Yu et al. (2013); Chen and Gridley (2013b); Menezes (2014); Frías et al. (2015); Xu et al. (2015); Saitoh et al. (2016); Wang et al. (2017a); Liu et al. (2018)
SNAI2	Niessen et al. (2008); Lambertini et al. (2010); Casas et al. (2011); Chen and Gridley (2013b); Cooley et al. (2014); Kumar et al. (2015); Welch-Reardon et al. (2015); Kobayashi and Ozawa (2018)
STAT3	Chen et al. (2008)
TGFB	van Meeteren and Ten Dijke (2012); Pardali et al. (2017)
TGFBR	Chen et al. (2012); van Meeteren and Ten Dijke (2012); Pardali et al. (2017)
TWIST1	Howe et al. (2003); Zhang et al. (2018)
VEGFA	Forsythe et al. (1996); Chang et al. (2004); Harper and Bates (2008); Kumar et al. (2014); Simons et al. (2016); Weinstein et al. (2017)
VEGFR2	Funahashi et al. (2010); Kanki et al. (2011); Murakami et al. (2011); Abedin et al. (2014); Simons et al. (2016); Weinstein et al. (2017); Liu et al. (2018)
WNT5b	Wu et al. (2012); Menezes (2014); Wang et al. (2017a)
WNT7a	Menezes (2014)
ZEB1	Sánchez-Tilló et al. (2011); Wels et al. (2011); Sanchez-Tillo et al. (2014); Kobayashi and Ozawa (2018)
ZEB2	Takkunen et al. (2006); Katoh and Katoh (2009); Grabitz and Duncan (2012); Zhao et al. (2014)

**Table 2:** The changes to the update rule components necessary in order to reach a fixed pattern of molecular activation for each expected cell type or behavior.

Modification	Reason or desired effect
In ECs, E47 should be absent so that TWIST1 inhibits the transcription of SNAI1	Otherwise TWIST1 would activate SNAI1 in Stalk ECs.
GATA2 must not activate the transcription of TWIST1 in ECs	Prevents TWIST1, SNAI1 and SNAI2 activation in Phalanx cells.
Both SNAI1 and GATA2 should be required to inhibit SNAI2 expression	to preserve SNAI2 expression in Stalk cells.

### Our Model Formalized as a Set of Boolean Equations

(1) AP1(t+1)=WNT5b(t)∨SMAD2(t)

(2) CTNNB(t+1)=WNT5b(t)∨WNT7a(t)

(3) DLL4(t+1)=DLL4(t)

(4) ETS1(t+1)=VEGF2(t)∨FGF2(t)∨ZEB2(t)∨AP1(t)

(5) FGF2(t+1)=FGF2(t)

(6) FLI1(t+1)=FLI1(t)∨GATA2(t)∨HIF1α(t)

(7) GATA2(t+1)=FLI1(t)∨GATA2(t)∨VEGFR2(t)

(8) HIF1α(t+1)=HIF1α(t)

(9) $$\begin{array}{ll} LEF1\left(t+1\right)= & LEF1\left(t\right)\vee NRARP\left(t\right)\vee SMAD2\left(t\right)\\ & \vee NF\kappa B\left(t\right)\vee CTNNB\left(t\right) \end{array}$$

(10) NFκB(t+1)=PDGF_AB(t)

(11) NOTCH(t+1)=DLL4(t)∧¬NRARP(t)

(12) NRARP(t+1)=NOTCH(t)

(13) NRP1(t+1)=VEGFA(t)∧(ETS1(t)∨GATA2(t))∧¬NOTCH(t)

(14) PDGF_AB(t+1)=PDGF_AB(t)

(15) SMAD1(t+1)=TGFβR(t)∧¬SMAD6(t)

(16) SMAD2(t+1)=TGFβR(t)∧¬SMAD6(t)∧NRP1(t)

(17) SMAD6(t+1)=FLI1(t)∧GATA2(t)∧(NOTCH(t)∨SMAD1(t))

(18) $$\begin{array}{ll} SNAI1\left(t+1\right)= & (HIF1\alpha \left(t\right)\vee STAT3\left(t\right)\vee CTNNB\left(t\right)\vee AP1\left(t\right)\\ & \vee NF\kappa B\left(t\right)\vee SMAD2\left(t\right))\\ & \wedge \left(NOTCH\left(t\right)\vee CTNNB\left(t\right)\right)\\ & \wedge \neg TWIST1\left(t\right)\wedge \neg SNAI1\left(t\right)\wedge \neg SNAI2\left(t\right) \end{array}$$

(19) $$\begin{array}{l} SNAI2(t+1)=\neg (SNAI1(t)\wedge GATA2(t))\\ \wedge (SMAD2(t)\vee SNAI2(t)\vee TWIST1(t)\vee LEF1(t)\vee NOTCH(t)) \end{array}$$

(20) STAT3(t+1)=VEGFR2(t)

(21) TGFβ(t+1)=TGFβ(t)

(22) TGFβR(t+1)=TGFβ(t)∧¬FGF2(t)

(23) TWIST1(t+1)=CTNNB(t)∨HIF1α(t)

(24) VEGFA(t+1)=(¬WNT5b(t)∧¬TGFβR(t)∧(STAT3(t)∨HIF1α(t)))∨VEGFA(t)

(25) $$\begin{array}{ll} VEGFR2\left(t+1\right)= & VEGFA\left(t\right)\wedge \neg SNAI1\left(t\right)\wedge \neg NOTCH\left(t\right)\\ & \wedge \left(FLI1\left(t\right)\vee GATA2\left(t\right)\vee ETS1\left(t\right)\right) \end{array}$$

(26) WNT5b(t+1)=WNT5b(t)

(27) WNT7a(t+1)=WNT7a(t)

(28) ZEB1(t+1)=SNAI2(t)∨CTNNB(t)∨LEF1(t)

(29) $$\begin{array}{ll} ZEB2\left(t+1\right)= & HIF1\alpha \left(t\right)\vee ETS1\left(t\right)\vee SMAD2\left(t\right)\\ & \vee AP1\left(t\right)\vee SNAI1\left(t\right) \end{array}$$

### Feedback Circuits

The regulatory network, as shown in **Figure 2**, contains a total of 74 feedback circuits. However, only 11 circuits are functional, eight of them positive and three negative (**Supplementary Table S1**). The three functional negative circuits are of particular importance because they originate the cyclic behavior in the dynamical model. Specifically, *a*) SNAI1 inhibits itself; *b*) NOTCH activates NRARP, which in turn inhibits NOTCH; and *c*) SMAD1 activates SMAD6, which inhibits SMAD1. Additionally, the microenvironment is defined by the pattern of activation of seven source molecules, and since there are possible microenvironments, the minimum number of attractors is 128. However, the simulated dynamic behavior results in 444 attractors due to the effect of the functional positive feedback circuits (Azpeitia et al., 2017; Rozum and Albert, 2018). This is in qualitative accordance with the large diversity of EC and MC patterns of molecular activation that has been reported in the literature (Chi et al., 2003; Ho et al., 2018).

### Fixed and Cyclic Patterns of Molecular Activation

**Figure 3:**
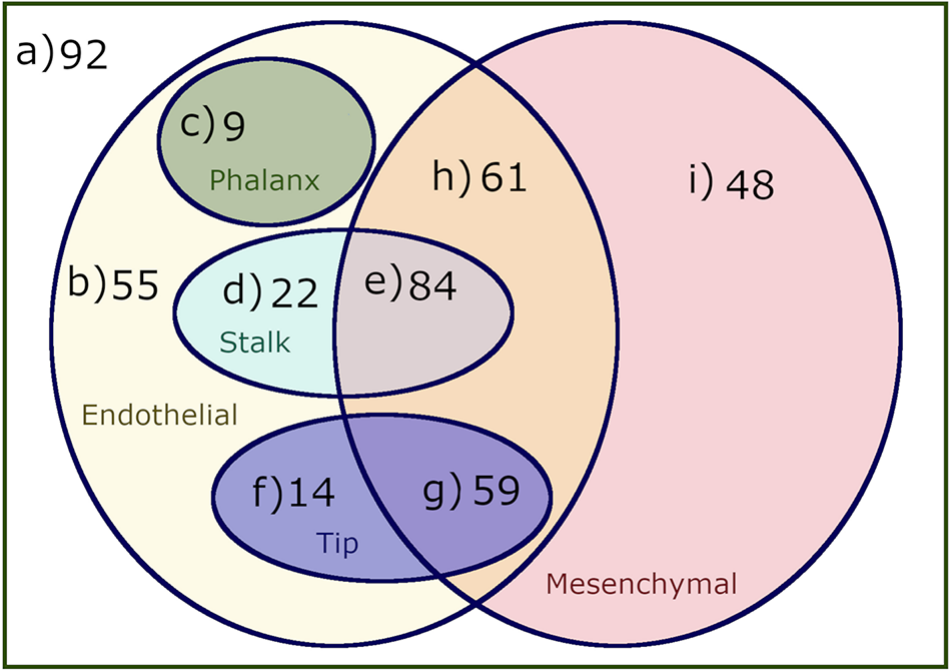
A venn diagram of the attractors reached by simulating the dynamic behavior of our Boolean model. We classified the attractors as mesenchymal, endothelial, phalanx, stalk, and tip cells, forming nine disjoint sets that represent the following cell types and behaviors: a) Cell types that are neither endothelial nor mesenchymal, b) Endothelial cell types that are not mesenchymal and do not behave as phalanx, stalk or tip cells, c) Endothelial and nonmesenchymal phalanx cell types, d) Endothelial and nonmesenchymal stalk cell types, e) Endothelial and mesenchymal stalk cell types, f) Endothelial and nonmesenchymal tip cell types, g) Mesenchymal and endothelial tip cell types, h) Endothelial and mesenchymal cell types that do not exhibit tip stalk or phalanx cell behavior, and i) Mesenchymal only cell types.

The analysis of the dynamical behavior of the model shows that the system has 444 attractors, 169 of which are fixed points, 18 are cyclic attractors of size 2, and 257 are cyclic attractors of size 4. These attractors correspond to stationary or cyclic patterns of molecular activation, which in turn can be identified with specific cell types and cellular states. Using the procedure described in Section 2.3, these attractors can be identified as belonging to Endothelial, Mesenchymal, Phalanx, Stalk, and Tip sets, which intersect each other but that can be dissected into nine disjoint sets, as shown in **Figure 3**. The specific active and inactive molecules for all these sets are shown in **Table 3**.

**Table 3:** Simulated cell type characteristics: Each cell type is represented by a group of attractors sorted as explained in *Molculear Pattern Identifcation*

Cell type	Active molecules	Inactive molecules	Fraction of the state space covered by the trap space
Non-EC and Non-MC	N.A.	AP1, FLI1, GATA2, HIF1*α*, NRP1, SMAD2, SMAD6, SNAI1, STAT3, VEGFR2, WNT5b	0.64206%
ECs	FLI1, GATA2	SNAI1	97.24696%
ECs only	FLI1, GATA2, SNAI2, ZEB1	CTNNB, HIF1*α*, TWIST1, WNT5b, WNT7a	7.12915%
MCs	ETS1, SNAI2, TWIST1, ZEB1, ZEB2	SNAI1	87.32046%
MCs only	CTNNB, ETS1, LEF1, SNAI2, TWIST1, ZEB1, ZEB2	FLI1, GATA2, HIF1*α*, NRP1, SMAD2, SMAD6, SNAI1, STAT3, VEGFA, VEGFR2	2.11098%
ECs and MCs	ETS1, FLI1, GATA2, SNAI2, TWIST1, ZEB1, ZEB2	SNAI1	85.20948%
ECs and MCs only	ETS1, FLI1, GATA2, SNAI2, TWIST1, ZEB1, ZEB2	SNAI1	29.79242%
Phalanx	FLI1, GATA2	AP1, CTNNB, DLL4, HIF1*α*, LEF1, NFkB, NOTCH, NRARP, NRP1, PDGF_AB, SMAD2, SNAI1, SNAI2, STAT3, TWIST1, VEGFA, VEGFR2, WNT5b, WNT7a, ZEB1	0.00322%
Stalk	CTNNB, FLI1, GATA2, LEF1, SNAI2, TWIST1, ZEB1	NRP1, SMAD2, SNAI1, STAT3, VEGFA, VEGFR2	26.56887%
Stalk MCs	CTNNB, ETS1, FLI1, GATA2, LEF1, SNAI2, TWIST1, ZEB1, ZEB2	NRP1, SMAD2, SNAI1, STAT3, VEGFA, VEGFR2	26.43057%
Stalk Non-MCs	CTNNB, FLI1, GATA2, LEF1, SNAI2, TWIST1, WNT7a, ZEB1	AP1, FGF2, HIF1*α*, NRP1, SMAD2, SNAI1, STAT3, VEGFA, VEGFR2, WNT5b	0.13830%
Tip	ETS1, FLI1, GATA2, NRP1, STAT3, VEGFA, VEGFR2, ZEB2	DLL4, NOTCH, NRARP, SNAI1	33.7533%
Tip MCs	ETS1, FLI1, GATA2, NRP1, SNAI2, STAT3, TWIST1, VEGFA, VEGFR2, ZEB1, ZEB2	DLL4, NOTCH, NRARP, SNAI1	28.98649%
Tip Non-MCs	ETS1, FLI1, GATA2, NRP1, STAT3, VEGFA, VEGFR2, ZEB2	CTNNB, DLL4, HIF1*α*, NOTCH, NRARP, SNAI1, TWIST1, WNT5b, WNT7a	4.76681%

The presence or absence of a seven ligands in the extracellular microenvironment together with the pattern of molecular activation within the cell define the attractor reached after a simulation of the dynamic behavior of our model. In order to illustrate how this process functions, we simulated the behavior of our model cell in an EndMT-inducing extracellular microenvironment where HIF1 and FGF2 are absent while DLL4, TGFB, WNT5b, WNT7a, and PDGF_AB are present. The attractors reached by our model under such conditions are shown in **Table 4**. Attractor 1 corresponds to the expected pattern of expression of a mesenchymal stalk cell. Note that here, FLI1 and GATA2 are active, and their activity is sustained by three positive feedback circuits. Attractor 2 represents the pattern of expression of an EC that competes with its neighbors to become a tip cell, and cannot fully become a tip cell due to the paracrine effect of the DLL4 ligand expressed by its neighbors (Jakobsson et al., 2010). Note that in Attractor 2, in addition to GATA2 and FLI1, VEGFA is active, and its activity is sustained by a positive feedback circuit. Attractor 3 represents a nonendothelial mesenchymal cell where FLI1, GATA2, and VEGFA are inactive.

**Table 4:** The attractors reached by our model in an endothelial-to-mesenchymal transition (EndMT)–inducing extracellular microenvironment where HIF1 and FGF2 are absent while DLL4, TGFB, WNT5b, WNT7a, and PDGF_AB are present.

Attractor	1	1	1	1	2	2	2	2	3	3	3	3
AP1	1	1	1	1	1	1	1	1	1	1	1	1
CTNNB	1	1	1	1	1	1	1	1	1	1	1	1
DLL4	1	1	1	1	1	1	1	1	1	1	1	1
ETS1	1	1	1	1	1	1	1	1	1	1	1	1
FGF2	0	0	0	0	0	0	0	0	0	0	0	0
FLI1	1	1	1	1	1	1	1	1	0	0	0	0
GATA2	1	1	1	1	1	1	1	1	0	0	0	0
HIF1a	0	0	0	0	0	0	0	0	0	0	0	0
LEF1	1	1	1	1	1	1	1	1	1	1	1	1
NF*κ*B	1	1	1	1	1	1	1	1	1	1	1	1
NOTCH	0	1	1	0	0	0	1	1	0	1	1	0
NRARP	0	0	1	1	1	0	0	1	0	0	1	1
NRP1	0	0	0	0	0	1	1	0	0	0	0	0
PDGF_AB	1	1	1	1	1	1	1	1	1	1	1	1
SMAD1	0	1	1	0	0	0	1	1	1	1	1	1
SMAD2	0	0	0	0	0	0	1	1	0	0	0	0
SMAD6	0	0	1	1	1	0	0	1	0	0	0	0
SNAI1	0	0	0	0	0	0	0	0	0	0	0	0
SNAI2	1	1	1	1	1	1	1	1	1	1	1	1
STAT3	0	0	0	0	0	0	1	1	0	0	0	0
TGFB	1	1	1	1	1	1	1	1	1	1	1	1
TGFBR	1	1	1	1	1	1	1	1	1	1	1	1
TWIST1	1	1	1	1	1	1	1	1	1	1	1	1
VEGFA	0	0	0	0	1	1	1	1	0	0	0	0
VEGFR2	0	0	0	0	0	1	1	0	0	0	0	0
WNT5b	1	1	1	1	1	1	1	1	1	1	1	1
WNT7a	1	1	1	1	1	1	1	1	1	1	1	1
ZEB1	1	1	1	1	1	1	1	1	1	1	1	1
ZEB2	1	1	1	1	1	1	1	1	1	1	1	1

Active molecules are shown in white, and inactive molecules are shown in gray.

### Robustness Analysis

The first type of robustness analysis was the evaluation of the effects on cell types and behaviors caused by the simulation of all possible single loss and gain-of-function mutations in the model. These are summarized in **Table 5**. Observe that only 24 of 58 possible single mutations do not alter the qualitative behavior of the model, as measured by the type of resulting attractors. The relative low robustness to gene mutations is likely to be due to the fact that we only included in our model molecules with an important biological role. Furthermore, the simulation of the other single mutations all results in the disappearance of certain cell types. However, according to our model, each cell type or behavior is very robust to single gain- and loss-of-function gene mutations. Notably, the larger the number of attractors classified as a cell type or behavior, the larger the robustness of the cell type is to gene mutations.

**Table 5:** The simulated single gain and loss-of-function mutations that affect each cell type.

Effect	Mutations	Robustness
Wild type	AP1−, DLL4−, FGF2−, HIF1a−, LEF1−, NFkB−, NOTCH−, NRARP−, PDGF_AB−, SMAD1−, SMAD1+, SMAD2−, SMAD6−, SMAD6+, SNAI1−, STAT3−, STAT3+, TGFB−, TGFB+, TGFBR−, TGFBR+, VEGFR2−, WNT5b−, ZEB1+	41.38%
Loss of nonendothlial and nonmesenchymal cells	FLI1+, GATA2+, HIF1a+, VEGFR2+, WNT5b+	91.38%
Loss of nonmesenchymal, nonphalanx, nontip, and nonstalk ECs	CTNNB+, FLI1−, GATA2−, HIF1a+, WNT5b+, WNT7a+	89.66%
Loss of phalanx cells	CTNNB+, DLL4+, FLI1−, GATA2−, HIF1a+, LEF1+, NFkB+, NOTCH+, NRARP+, NRP1+, PDGF_AB+, SMAD2+, SNAI1+, SNAI2+, TWIST1+, VEGFA+, WNT5b+, WNT7a+	68.97%
Loss of nonmesenchymal stalk cells	AP1+, CTNNB−, ETS1+, FGF2+, FLI1−, GATA2−, HIF1a+, NRP1+, SMAD2+, SNAI1+, SNAI2−, VEGFA+, VEGFR2+, WNT5b+, WNT7a−, ZEB2+	72.41%
Loss of mesenchymal stalk cells	CTNNB−, FLI1−, GATA2−, NRP1+, SNAI1+, SNAI2−, TWIST1−, VEGFA+, ZEB1−, ZEB2−	82.76%
Loss of nonmesenchymal tip cells	CTNNB+, DLL4+, ETS1−, FLI1−, GATA2−, HIF1a+, NOTCH+, NRP1−, TWIST1+, VEGFA−, WNT5b+, WNT7a+	79.31%
Loss of mesenchymal tip cells	DLL4+, ETS1−, FLI1−, GATA2−, NOTCH+, NRP1−, SNAI2−, TWIST1−, VEGFA−, ZEB1−, ZEB2−	81.03%
Loss of mesenchymal ECs that are neither phalanx, tip nor stalk cells	FLI1−, GATA2−, NRP1+, SNAI2−, TWIST1−, ZEB1−, ZEB2−	87.93%
Loss of nonendothelial mesenchymal cells	CTNNB−, FLI1+, GATA2+, HIF1a+, SNAI2−, TWIST1−, VEGFA+, VEGFR2+, ZEB1−, ZEB2−	82.76%

**Figure 4:**
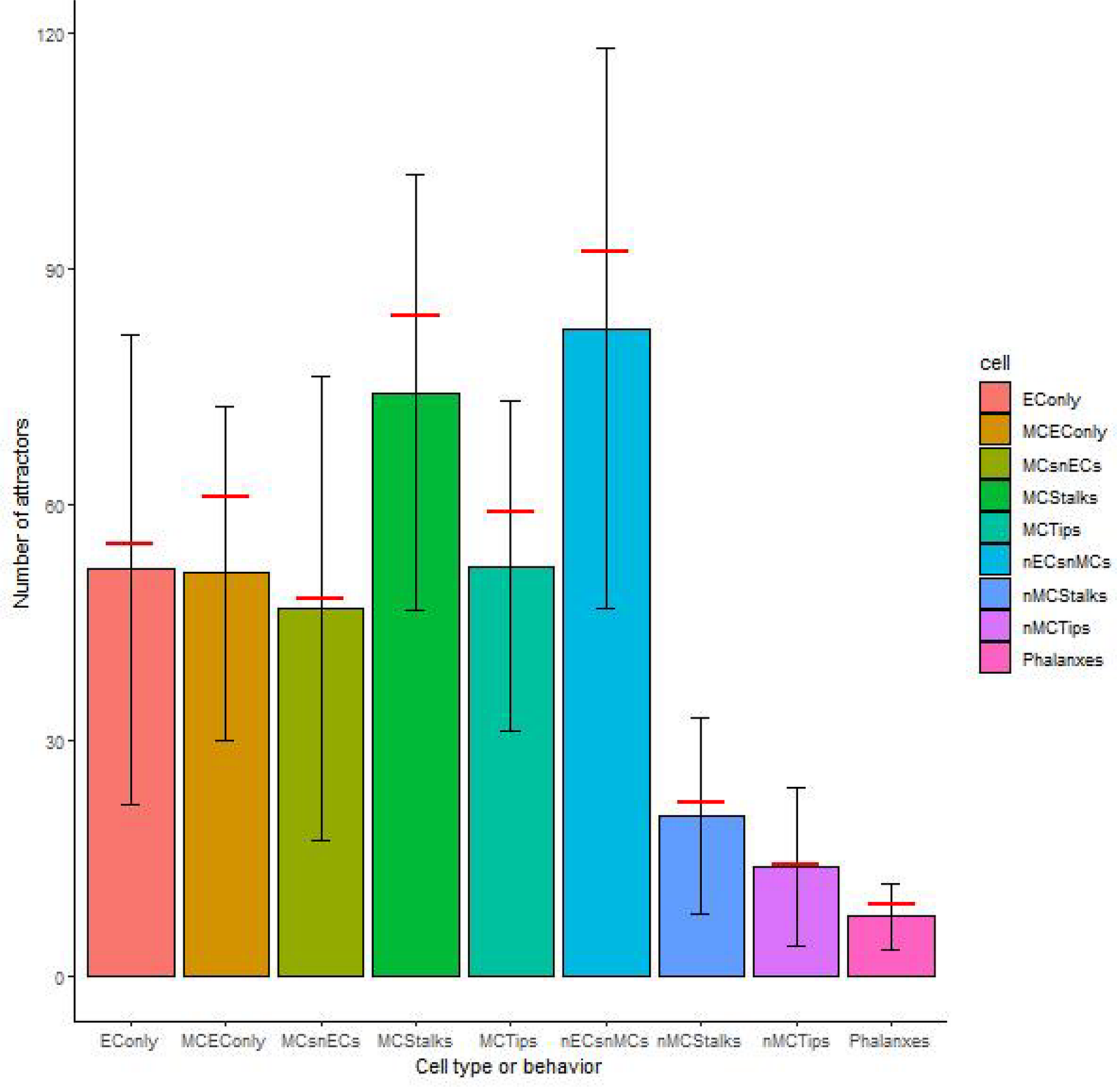
The robustness of the cell types and behaviors to changes in the update rule: The height of the bars represents the median number of attractors of each cell type or behavior, the error bars represent one standard deviation over and under the mean respectively, and the red horizontal line segments represent the number of attractors of each cell type or behavior on our original model.

As for the robustness of each cell type against noise in the update rule, in all cases, the original model reached slightly more attractors than the mean of the 100 000 networks with perturbed update rules, as shown in **Figure 4**. Observe that the standard deviation in the number of attractors for all cell types and behaviors is relatively large, and therefore the robustness of the number of attractors that represent each cell type or behavior is low. The maximum numbers of attractors for each of the cell types were the following: nECsnMCs 238, EConly 243, Phalanxes 27, nMCStalks 106, MCStalks 216, nMCTips 70, MCTips 149, MCEConly 129, MCsnECs 237, while the minimum values reached 0 for all cell types and behaviors.

**Figure 5:**
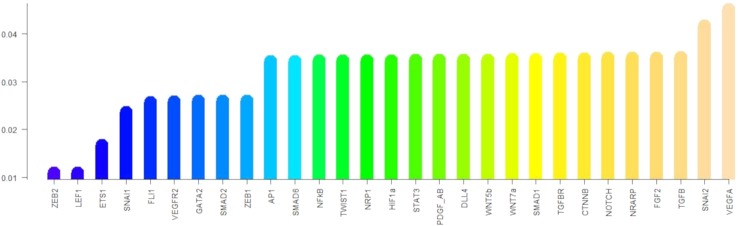
The sensitivity of each component of the update rule: The height of the bars represents the sensitivity of the components of the update rule to perturbations that affect one node.

**Figure 6:**
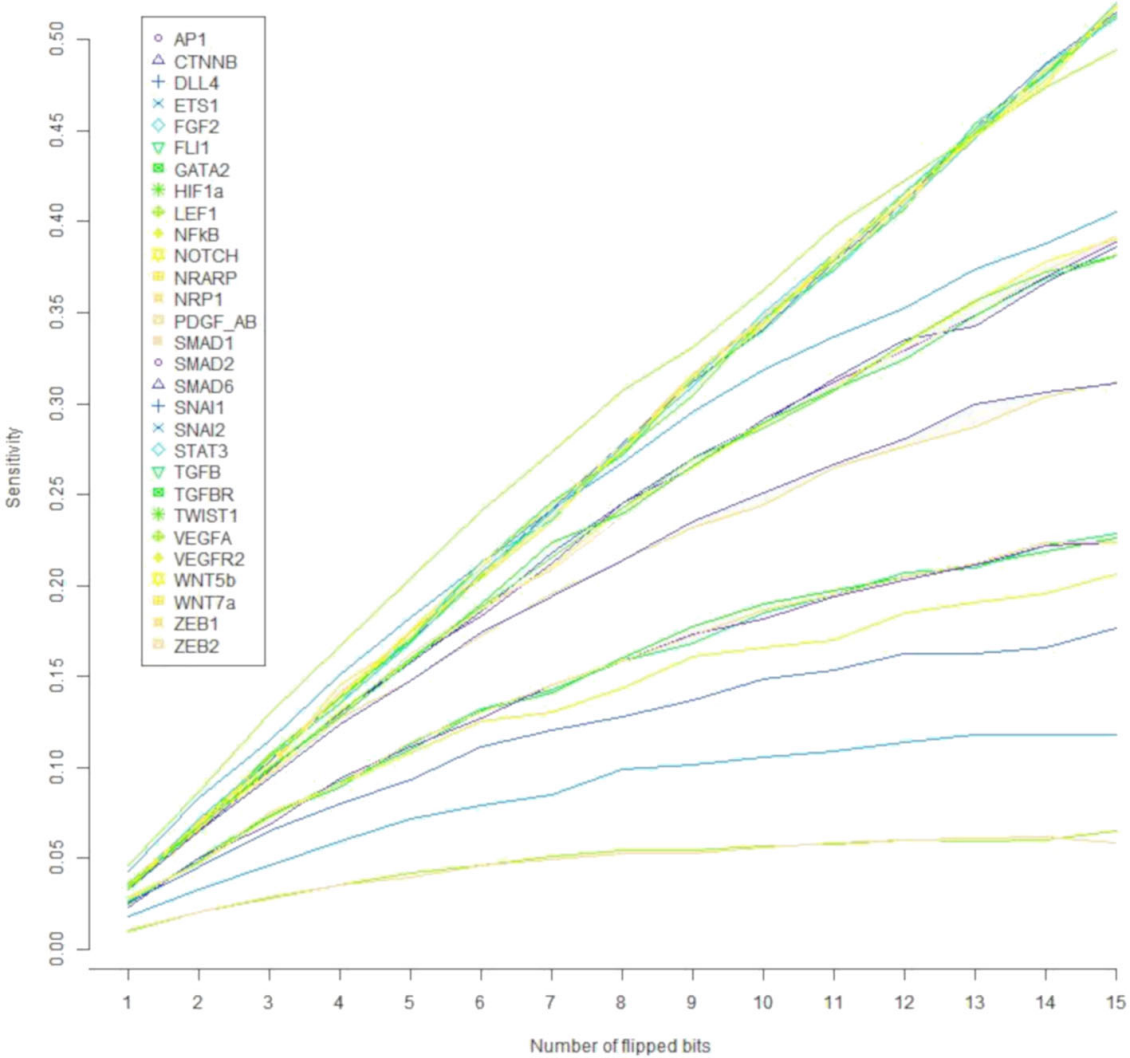
The effect of the number of flipped bits on the sensitivity of the update rule components. Note that the components segregate according to their sensitivity to molecular activation noise into five categories.

Regarding the robustness of the components of the update rule to noise in the activation value, all such components are sensitive to less than 4.6% of all single bit perturbations that are the most likely to occur, as shown in **Figure 5**. Notice that the components corresponding to ZEB2, LEF1, and ETS1 are under 2%; SNAI1, FLI1, VEGFR2, GATA2, SMAD2, and ZEB1 have a sensitivity of about 2.5%, while most of the other components have a sensitivity between 3.4% and 3.5% except for SNAI2 and VEGFA that have a sensitivity of over 4%. Nonetheless, the sensitivity of all the components increases as the number of flipped bits increases (**Figure 6**). When the activity of 15 nodes is affected, the components can be grouped by their sensitivity into 5 categories: *1)* VEGFA, STAT3, NRARP, FGF2, PDGF_AB, HIF1a, DLL4, WNT7a, WNT5b, and NF*κ*B, TGF*β* with a sensitivity between 49.4% and 52%. *2)* TGFBR, TWIST1, CTNNB, AP1, NOTCH, SMAD1, and SNAI2 with a sensitivity between 38.1% and 40.55%. *3)* SMAD6, and NRP1 with a sensitivity of 31.1%, and 31.2% respectively. *4)* SNAI1, VEGFR2, SMAD2, ZEB1, GATA2, and FLI1 with a sensitivity between 17.7% and 22.8%. And *5)* ETS1, LEF1, and ZEB2 with a sensitivity between 5.9% and 11.8%. Note that there exists a trend that is independent of the number of flipped bits, where the sensitivity for the components that represent ligands that define the extracellular microenvironment is high, and the sensitivity of the components that represent molecules used as cell type markers is low. The very low sensitivity of the components that represent ETS1, LEF1, and ZEB2 is in accordance with the importance that of the three transcription factors not only during EndMT, but also during other cell differentiation processes. Specifically, ZEB2 is involved in T cell differentiation (Goossens et al., 2019) and neurological development (Epifanova et al., 2018). LEF1 is important during osteogenesis (Li et al., 2018), immune cell regulation (Chae and Bothwell, 2018), and hair follicle development (Abaci et al., 2018). ETS1 is an important regulator of lymphatic cell differentiation and physiology (Garrett-Sinha et al., 2016).

**Figure 7:**
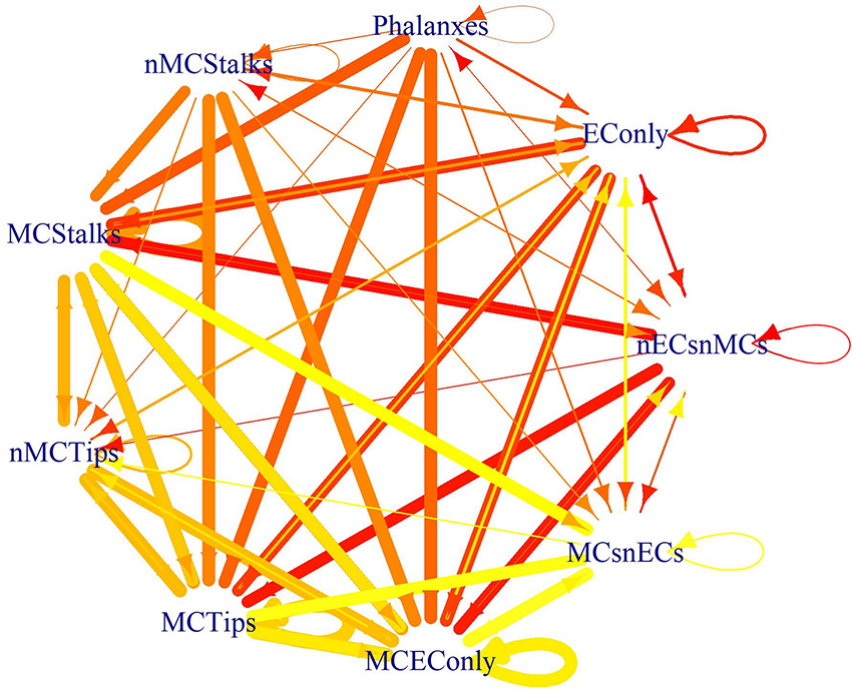
The effect of the perturbations as a state transition graph: The width of the edges represents the fraction of the perturbations that lead to that transition, and the color of the edge denotes the original cell type or behavior.

Finally, one of the goals of this modeling effort was to understand the conditions that cause an EC cell to change, either by behaving differently or by differentiating partially or fully into a mesenchymal cell. Further, EndMT is a gradual, and reversible process and therefore we also aimed to fathom the conditions that cause MEnT. Moreover, the intermediate states reached through partial-EndMT are important due to their physiological role during sprouting angiogenesis (Welch-Reardon et al., 2014), and due to the similarity between EndMT and EMT; it seems likely that the intermediate states are also important from a dynamic perspective (Lu et al., 2013; Li et al., 2016). In order to grasp the conditions that lead to EndMT and MEnT, for each cyclic or fixed pattern of molecular activation of our model, we simulated all possible perturbations in the molecules that are either microenviromental signals or the main transcription factors involved in the regulation of EC identity. Specifically DLL4, FGF2, FLI1, GATA2, HIF1*α*, PDGF_AB, TGF*β*, VEGFA, WNT5b, and WNT7a. The possible effects of the 1024 perturbations are available for the interested reader as the 81 **Supplementary Files** in the folder https://github.com/NathanWeinstein/EndMT/T_Results.zip in the format used to export R objects, namely,. RData and are summarized in **Table 6** which can be interpreted as a cell type or behavior transition graph (**Figure 7**).

**Table 6:** The number and the characteristics of the pertubations in the activation state of the molecules DLL4, FGF2, FLI1, GATA2, HIF1*α*, PDGF_AB, TGF*β*, VEGFA, WNT5b, and WNT7a that cause cell type or cell behavior changes: Each cell contains first the number of perturbations that trigger the transitions, if the number is bigger than 0, the cell contains the molecules that are active in all perturbations +(), as well as the molecules that are inactive in all perturbations −().

	nECsnMCs	EConly	Phalanxes	nMCStalks	MCStalks	nMCTips	MCTips	MCEConly	MCsnECs
nECsnMCs	40, −(FLI1, GATA2, HIF1a, WNT5b)	80, −(HIF1a, WNT5b, WNT7a)	12, −(DLL4, HIF1a, PDGF_AB, VEGFA, WNT5b, WNT7a)	24, +(WNT7a) −(FGF2, HIF1a, VEGFA, WNT5b)	288, −(VEGFA)	32, +(VEGFA) −(DLL4, HIF1a, WNT5b, WNT7a)	288, −(DLL4)	296	48, -(FLI1, GATA2, HIF1a, VEGFA)
EConly	40, −(FLI1, GATA2, HIF1a, WNT5b)	90, −(HIF1a, WNT5b, WNT7a)	0	24, + (WNT7a) −(FGF2, HIF1a, VEGFA, WNT5b)	324, −(VEGFA)	64, −(DLL4, HIF1a, WNT5b, WNT7a)	320, −(DLL4)	328	48, -(FLI1, GATA2, HIF1a, VEGFA)
Phalanxes	30, −(FLI1, GATA2, HIF1a, WNT5b)	68, −(HIF1a, WNT5b, WNT7a)	12, −(DLL4, HIF1a, PDGF_AB, VEGFA, WNT5b, WNT7a)	24, +(WNT7a) −(FGF2, HIF1a, VEGFA, WNT5b)	288, −(VEGFA)	32, +(VEGFA) −(DLL4, HIF1a, WNT5b, WNT7a)	288, −(DLL4)	296	48, −(FLI1, GATA2, HIF1a, VEGFA)
nMCStalks	40, −(FLI1, GATA2, HIF1a, WNT5b)	80, −(HIF1a, WNT5b, WNT7a)	0	24, +(WNT7a) −(FGF2, HIF1a, VEGFA, WNT5b)	264, −(VEGFA)	32, +(VEGFA) −(DLL4, HIF1a, WNT5b, WNT7a)	288, −(DLL4)	296	40, −(FLI1, GATA2, HIF1a, VEGFA)
MCStalks	16, −(FLI1, GATA2, HIF1a, VEGFA, WNT5b, WNT7a)	80, −(HIF1a, WNT5b, WNT7a)	0	0	288, −(VEGFA)	32, +(VEGFA) −(DLL4, HIF1a, WNT5b, WNT7a)	288, −(DLL4)	296	48, −(FLI1, GATA2, HIF1a, VEGFA)
nMCTips	0	72, −(HIF1a, WNT5b, WNT7a)	0	0	288, −(VEGFA)	64, −(DLL4, HIF1a, WNT5b, WNT7a)	320, −(DLL4)	328	0
MCTips	0	72, −(HIF1a, WNT5b, WNT7a)	0	0	288, −(VEGFA)	64, −(DLL4, HIF1a, WNT5b, WNT7a)	320, −(DLL4)	328	0
MCEConly	16, −(FLI1, GATA2, HIF1a, VEGFA, WNT5b, WNT7a)	90, −(HIF1a, WNT5b, WNT7a)	0	0	324, −(VEGFA)	64, −(DLL4, HIF1a, WNT5b, WNT7a)	320, −(DLL4)	328	48, −(FLI1, GATA2, HIF1a, VEGFA)
MCsnECs	16, −(FLI1, GATA2, HIF1a, VEGFA, WNT5b, WNT7a)	80, −(HIF1a, WNT5b, WNT7a)	0	0	288, −(VEGFA)	32, +(VEGFA) −(DLL4, HIF1a, WNT5b, WNT7a)	288, −(DLL4)	296	48, −(FLI1, GATA2, HIF1a, VEGFA)

The perturbations that do not cause a change in cell type or behavior are shown in white, those that cause a full endothelial-to-mesenchymal transition (EndMT) transition are shaded in dark cyan, those that represent a partial EdnMT are shaded in medium cyan, those that represent an endothelial activation are shown in light cyan, those that represent an endothelial deactivation are shown in light amber, those that represent a partial mesenchymal-to-endothelial transition are shown in amber, full mesenchymal-to-endothelial transitions are shown in dark amber, and other perturbations are shown in gray.

### Model Validation

An exhaustive comparison between the global effect of all possible single gene mutations in the model and the reported experimental results are presented in **Supplementary Table S2**, and summarized in **Table 7**. Overall, the behavior of the model is very good at recovering the effect of a large proportion of the reported mutants. Notice that several of the discrepancies are because the model does not incorporate multicellular or morphological effects, or because the reported effects involve some molecules not included in the model. This is encouraging given the qualitative nature of the model. Of the 58 possible mutations, we successfully simulated the specific effects reported for 37 (63.8%) of them. Furthermore, the effects of 4 (6.9%) mutations constitute predictions of our model. 13 (22.4%) mutations cause multicellular effects that we could not reproduce using our model. Two mutations (3.45%) cause morphological changes in the shapes of cells that are also beyond the scope of our model. 4 (6.9%) mutations affect molecules that we did not include in the model. 4 (6.9%) mutations have an effect that was only observed in lymphatic ECs. Some of the reported effect of 7 (12.1%) mutations was not recovered by the simulated behavior of our model. For two mutations (3.45%), there are conflicting effects reported in the literature.

**Table 7:** The capacity of our model to simulate the effects of mutations as reported in the literature.

Successfully simulated	CTNNB+, DLL4+, ETS1−, FGF2−, FLI1−, FLI1+, GATA2−, HIF1*α*−, HIF1**α**+, LEF1+, NF*κ*B+, NOTCH1−, NOTCH+, NRARP+, PDGF_AB−, PDGF_AB+, SMAD6−, SMAD6+, SNAI1+, SNAI2−, SNAI2+, STAT3−, TGF*β*−, TGF*β*+, TGF*β*R−, TGF*β*R+, TWIST1−TWIST1+, VEGFA−WNT5b−WNT5b+, WNT7a+, ZEB1−, ZEB2−, ZEB2+.
Affect the likelihood of transient patterns of expression	AP1−, NF*κ*B−, STAT3+.
Predictions of our model	AP1+, GATA2+, SMAD1+, SMAD2+.
Unable to simulate the multicellular effect	CTNNB−, DLL4−, DLL4−, LEF1−, NRARP−, SMAD6−, SNAI2−, SNAI2+, VEGFA+, VEGFR2−, VEGFR2+, WNT7a−, ZEB2−.
Unable to simulate the morphological cell change	SNAI1−, SNAI1+
Affects molecules not included in our model	ETS1+, SNAI2−, SNAI2+, ZEB2+
Some effects were only observed in lymphatic ECs	FGF2−, FGF2+, WNT5b−, WNT5b+
Some effects not simulated	SMAD1−, SMAD2−, STAT3+, TGF*β*R+, TWIST1+, VEGFA+, ZEB1+
Conflicting effects reported in the literature	NRP1−, NRP1+

#### Simulating EC Behavior and Differentiation During Developmental Processes and the Progression of Diseases Related to EndMT

During early heart valve formation, *embryonic heart cushion EndMT* is triggered by TGF, WNT, and NOTCH signaling and is inhibited by VEGF signaling (von Gise and Pu, 2012). This behavior is recovered by the simulated dynamic behavior of our model, (TGF*β*+, WNT5b+, and NOTCH+ increase the fraction of mesenchymal attractors, and VEGFA+ prevents full-EndMT). TGF*β*2−, ALK1−, ALK5−, SMAD4−, SMAD6+, NOTCH1−, VEGFA+, CTNNB−, and PDGF_AB− inhibit EndMT, and cause endocardial cushion hypoplasia. In contrast SMAD6- causes heart valve hyperplasia by increasing the number of MCs (von Gise and Pu, 2012). According to the simulated dynamic behavior of our model, the simulated loss of TGF*β*, which represents TGF-*β*2, and the loss of TGF*β*R, which represents all TGF receptors including ALK1 and ALK2, reduces the fraction of mesenchymal attractors. Further, the loss of the cofactor SMAD4 can be simulated as the loss of both SMAD1 and SMAD2 function and does not affect the fraction of mesenchymal attractors. The simulated SMAD6 gain of function mutation also does not affect the fraction of mesenchymal attractors. Simulated NOTCH loss of function reduces the fraction of mesenchymal attractors. Moreover, simulated VEGFA gain of function and CTNNB loss of function prevent the existence of nonendothelial mesenchymal attractors. Additionally, the simulated loss of PDGF_AB reduces the fraction of mesenchymal attractors. Lastly, the simulated loss of SMAD6 function exhibits a slight increase in the fraction of mesenchymal attractors.

The initial stages of *atherosclerosis and vascular calcification* are characterized by neointimal hyperplasia. Local disparity in shear stress is associated to neointimal lesions. While most neointimal cells originate from smooth muscle, some neointimal cells might arise from ECs that undergo EndMT. ECs that are exposed to disturbed flow undergo EndMT; conversely, uniform laminar shear stress hinders EndMT through KLF2, KLF4, MEK5, and ERK5 (Moonen et al., 2015). Molecularly, ERK5 is the main mitogen-activated protein kinase (MAPK) involved in the regulation of cardiovascular development (Nishimoto and Nishida, 2006), and VEGF/MAPK signaling activates the transcription of several transcription factors from the ETS family including *ets1* and *fli1* (Wythe et al., 2013). Furthermore, the low shear stress caused by disturbed nonlaminar flow at the sites where neointimal hyperplasia occurs leads to a decrease in *ets1*, and *fli1* expression. Therefore, we can simulate uniform laminar shear stress as the double gain of function mutation *ets1+/fli1+*, and a disturbed nonlaminar flow as the double mutant *ets1−/fli1−*. The simulated effect of *ets1−/fli1−* is the loss of all endothelial attractors, the number of nonendothelial mesenchymal attractors increases from 48 to 347, and the fraction of nonendothelial mesenchymal attractors increases from 0.108 to 0.69. This behavior can be interpreted as an increase in full EndMT resulting from nonlaminar flow. Moreover, the simulated effect of *ets1+/fli1+* is the loss of all nonendothelial mesenchymal attractors as well as an increase in the fraction of mesenchymal attractors from 0.567 to 0.816. This behavior can be interpreted as an increase in partial-EndMT and a complete inhibition of full EndMT. Therefore, according to our model, nonlaminar flow triggers full EndMT, and uniform laminar shear stress prevents full EndMT and upregulates angiogenesis-related partial EndMT. These results are in direct correspondence with the observed effect of uniform laminar and disturbed nonlaminar flow (Wragg et al., 2014).

Another important EndMT-related disease is *pulmonary arterial hypertension*, which is defined as a sustained pulmonary arterial pressure of more than 25 mm Hg at rest or more than 30 mm Hg during exercise, with a left ventricular pressure at the end of the diastole and a mean pulmonary-capillary wedge pressure lower than 15 mm Hg. The lung tissue of patients affected with pulmonary arterial hypertension is characterized by increased medial thickness, intimal fibrosis, plexiform lesions, and pulmonary arteriolar occlusion (Farber and Loscalzo, 2004). EndMT is involved in many of the pathological mechanisms associated with pulmonary arterial hypertension (Kovacic et al., 2019). At the molecular level, most cases of heritable pulmonary arterial hypertension involve mutations that affect the bone morphogenic protein (BMP) branch of the TGF signalling pathway including *ACVRL1(ALK1), BMPRII, ENG, SMAD1, SMAD4*, and *SMAD9*. Furthermore, BMPRII siRNA increases the expression of SNAI2 (Hopper et al., 2016). According to our model, the simulated gain of function mutation for SNAI2 increases the fraction of mesenchymal attractors, which is consistent with the experimental evidence.

Finally, hypoxia-induced EndMT is another important mechanism involved in the patophysiology of pulmonary arterial hypertension. HIF-1*α* directly binds to the promoter of TWIST1 and activates its expression (Zhang et al, 2018). Pulmonary arterial hypertension patients exhibit high levels of the cytokines IL-1*β* and TNF*α* that in the presence of TGF*β* induce EndMT in pulmonary arterial ECs *in vitro* (Good et al., 2015). IL-1*β* and TNF*α* induce EndMT by stabilizing NF-*κ*B (Sánchez-Duffhues et al., 2018). According to the simulated dynamic behavior of our model, a gain-of-function mutation of NF -*κ*B induces EndMT and elevates the expression of ZEB2.

## Discussion

### The Model as Theoretical Framework

Our model of the molecular regulatory network involved in the control of EndMT and EC activation integrates a vast amount of published experimental results. Therefore, our model constitutes a theoretical framework that summarizes the current knowledge and allows for the simulation of experiments that explore the molecular mechanisms involved in the regulation of EndMT *in silico*.

Many important questions about EndMT remain unanswered (Welch-Reardon et al., 2015). While such questions require a experimental approach to obtain a conclusive answer, models like the one presented here can be used to generate hypotheses to direct, or at least restrict, all the possible venues of experimental inquiry. In this sense, our model provides an important theoretical framework to understand the regulatory mechanisms behind EndMT. The following paragraphs provide testable hypotheses on some key aspects, according to our model.

*Are SNAI1, SNAI2, TWIST1, ZEB1, and ZEB2 all required for EndMT*? According to the dynamic behavior of the model, the loss of any of the transcription factors SNAI2, TWIST1, ZEB1, and ZEB2 prevents mesenchymal cell differentiation. Experimentally, the loss of SNAI2 (Niessen et al., 2008), TWIST1 (Mammoto et al., 2018), or ZEB1 (Sanchez-Tillo et al., 2010) prevents EndMT. ZEB2 has many functions in addition to its role during EndMT, its loss causes severe neurodevelopmental defects and cardiovascular malformations (Epifanova et al., 2018), while its specific effect during EndMT still needs to be elucidated. Conversely, the gain of ZEB2 function is sufficient to trigger EndMT (DaSilva-Arnold et al., 2018). SNAI1 over-expression can rescue the heart valve defects caused by the loss of SNAI2 (Niessen et al., 2008). According to our model, SNAI1 gain-of-function increases the fraction of mesenchymal attractors. This implies that it can trigger EndMT under certain circumstances. However, it cannot replace SNAI2 in fixed or cyclic patterns of expression because it inhibits its own expression.

Do SNAI1, SNAI2, TWIST1, ZEB1, and ZEB2 work sequentially, in parallel or in feedback circuits? TWIST1 regulates the transcription of both SNAI1 and SNAI2 (Yu et al., 2013; Forghanifard et al., 2017), while these two inhibit each other (Peiro et al., 2006; Chen and Gridley, 2013b). Then, SNAI1 activates the transcription of ZEB2 (Guaita et al., 2002; Takkunen et al., 2006), and SNAI2 activates the transcription of ZEB1 (Wels et al., 2011). The regulatory network presented here shows that SNAI1 and SNAI2 form part of several other circuits, including two functional feedback circuits where SNAI1 inhibits its own expression and SNAI2 activates its own expression. Furthermore, TWIST1, ZEB1, and ZEB2 appear to work sequentially with SNAI1 and SNAI2. In this context, our model contributes to the unraveling of several molecular circuits relevant for EndMT.

What regulates the expression of EndMT-promoting transcription factors? According to experimental observations (Piera-Velazquez and Jimenez, 2019) captured by our model, nonlaminar blood flow, inflammation, as well as TGF, WNT and NOTCH signaling pathway activity can trigger EndMT. By contrast, laminar blood flow, hypoxia, and VEGF signaling can inhibit full EndMT. Other molecular mechanisms that have been reported to regulate EndMT include the autocrine TGF activation by ET-1, the most potent known endogenous vasoconstrictor polypeptide that triggers EndMT. CAV-1, the main protein component of caveolae, is an important inhibitor of EndMT, by means of the internalization, trafficking, and degradation of TGF receptors. H_2_O_2_-induced oxidative stress, NOX2 and NOX4 can induce EndMT *via* TGF signaling. Fatty acid oxidation inhibits EndMT by activating SMAD7 and inhibiting TGF signaling. Hyperglycemia leads to EndMT through increased phosphorylation of ERK1/2, Angiotensin II synthesis, miR-200b and miR-328 upregulation, and ROCK1 activation (Piera-Velazquez and Jimenez, 2019). The wide variety in the patterns of expression that represent each cell type or behavior prevents the specification of the molecules that regulate EndMT. However, if the initial cell type or behavior is known, our model allows the specification of all possible perturbations that might cause a partial or full EndMT. This information is available in the **Supplementary Files** in the folder https://github.com/NathanWeinstein/EndMT/T_Results.zip in the format used to export R objects (.RData), and summarized in **Table 6** and **Figure 7**.

What controls whether cells undergo a full or partial EndMT? Many of the molecular mechanisms involved in the regulation of EndMT and angiogenesis remain unknown. Nevertheless, we know that the activity of several molecules, including NRP1 (Oh et al., 2002; Matkar et al., 2016), SNAI1 (Sun et al., 2018), SNAI2 (Welch-Reardon et al., 2015),n WNT5b (Wang et al., 2017a), and WNT7a (Howe et al., 2003; Pahnke et al., 2016) induce both EC activation and EndMT. Furthermore, TWIST1 (Mammoto et al., 2018), ZEB1 (Sanchez-Tillo et al., 2010), and ZEB2 (DaSilva-Arnold et al., 2018) induce EndMT and are not known to be involved in EC activation during angiogenesis. Finally, the activity of FGF2 (Ichise et al., 2014; Yang et al., 2015), and VEGFA (Paruchuri et al., 2006) induce angiogenesis and inhibit full EndMT. According to our model, all the cases that achieve a full EndMT with the loss of EC identity require FLI1, GATA2, HIF1*α*, as well as VEGFA inactivity. These molecules, as a group, have not been involved in this process up to now. In this case, our model serves as a guide to study the role of specific molecules, while at the same time providing a hypothesis of its role in the regulatory network.

### The Endothelial-to-Mesenchymal Transition in Medicine

EndMT is necessary during embryonic development for heart septation and heart valve morphogenesis. During the span of human life, EndMT is required to maintain heart valve homeostasis and adapt to hemodynamical changes. EndMT deregulation is involved in the pathophysiology of vascular malformation, vascular calcification, pulmonary arterial hypertension, and organ fibrosis (Medici, 2016; Sánchez-Duffhues et al., 2018).

EndMT is critical during the formation of the heart. Human heart development begins with the aggregation of splanchnopleuric mesenchyme cells that form part of the mesoderm into two endocardial tubes in the cardiogenic area of the embryo. Then, the two endocardial tubes fuse to form the primitive heart tube, which then begins to beat. After that, cardiac looping occurs. Next, septation and valve formation transpires (Moorman et al., 2003). Heart valves develop from endocardial cushions through two processes: the deposition of a special kind of ECM called cardiac jelly, and the arrival of mesenchymal cells that are the precursors to valve cells. Most cushion mesenchymal cells are derived from endocardial cells that undergo EndMT, while the rest originate from epicardium and cardiac neural crest cells that undergo EMT (MacGrogan et al., 2014). EndMT is also involved in adult valve homeostasis and disease. Adult heart valves are covered by a layer of ECs that undergo EndMT to replenish valve interstitial cells. Further, mechanical stretch-induced EndMT allows heart valves to adapt to changes in blood flow within the heart. However, excessive EndMT causes heart valve dysfunction thorough thickening or calcification. For instance, excessive EndMT after myocardial infraction can lead to mitral valve leaflet thickening and mitral regurgitation (Bischoff, 2019).

The formation and progression of *arteriovenous malformations and cerebral cavernous malformations* involves EndMT. CCM1, CCM2, and CCM3 loss-of-function mutations cause the formation of cerebral cavernous malformations. EC-specific disruption of the *Ccm1* causes TGF-mediated EndMT. Inhibiting TGF signaling reduces the number and size of vascular lesions caused by CCM1- deficiency (Maddaluno et al., 2013). Arteriovenous malformations are shunts that directly connect the afferent arteries to efferent veins, bypassing the usual capillary network. In addition to the fact that they take a large volume of space and prevent normal tissue perfusion, the nidus of arteriovenous malformations is prone to leaking or bursting, often causing unbearable pain and serious damage. ECs within brain arteriovenous malformations in mice undergo SOX2, and JMJD5-mediated EndMT that can be suppressed using *Pronethalol hydrochloride* (Yao et al., 2019).

Fibrosis is a wound healing process that involves the synthesis and accumulation of ECM proteins. Excessive fibrosis can cause functional organ failure. Myofibroblasts are the essential cell type in the pathogenesis of fibrotic disorders. In systemic sclerosis, cardiac fibrosis, renal fibrosis, idiopathic portal hypertension, colitis, and inflammatory bowel disease, some myofibroblasts express EC markers, suggesting that they originate from ECs that underwent TGF-induced EndMT (Pardali et al., 2017; Sánchez-Duffhues et al., 2018).

ECs can be found in every major organ in the body, and thorough EndMT ECs can become MCs that are capable of differentiating into pericytes, smooth muscle cells, skeletal muscle cells, cardiomyocytes, myofibroblasts, chondrocytes, osteocytes, adipocytes, hematopoietic stem cells, and other organ-specific cell types. Therefore, EndMT has vast potential for tissue engineering and regenerative medicine (Medici, 2016; Man et al., 2019). Currently, EndMT is harnessed to manage ECM production and remodeling during cardiovascular tissue graft engineering (Muylaert et al., 2015).

### Beyond a Synchronous BN

Despite the valuable insights provided by a Boolean model into the molecular mechanisms behind EndMT, it is evident that the complexity of the biological systems requires the incorporation of several characteristics. These constitute a set of improvements that will be incorporated into future versions of the model. The first improvement would be to convert the model into a continuous dynamical system, which will allow us to explore the biological relevance of the cyclic attractors reached by model, thus eliminating possible methodological artifacts caused by the synchronous update. Specifically, it is possible that some cycles found in the Boolean models might correspond to fixed point attractors with intermediate values when modeled as a continuous system. Furthermore, another important improvement would be the explicit modeling of the three-dimensional shape of the cells by specifying the cytoskeleton and cellular matrix. This information would allow the analysis of those signals that trigger EC cytoskeleton and ECM remodeling. This characteristic is important to understand the mechanism by which the shear stress caused by blood flow causes actin fibers within an EC to align with the flow (Kroon et al., 2017).

### Conclusion

We found sufficient information obtained from published experimental results to assemble a functional model of the molecular regulatory network involved in EndMT regulation. Therefore, everything indicates that sufficient main signaling pathways that regulate EndMT are already characterized. The next logical step is to unravel the operation of the molecular regulatory network involved in EndMT control at a systemic level. The model we describe in the present manuscript constitutes an initial qualitative analysis in that direction. EndMT is required for heart valve formation during embryonic development and is an important component in the pathophysioloy of cardiovascular and fibrotic diseases. Understanding how to regulate EndMT has vast applications in the treatment of disease and regenerative medicine. The simulated dynamic behavior of our model recovers fixed and cyclic patterns of molecular activation that correspond to the main cell types and behaviors involved in EndMT. Further, the simulated effect of most single gain and loss-of-function mutations of the molecules included in our model corresponds to the experimentally observed effect of the same mutations. Additionally, we used all possible perturbation patterns for 10 molecules to explore the conditions that cause EC activation, EndMT, and the reverse transitions. Based on the results of the perturbation analysis, we infer that the Phalanx and nonmesenchymal Stalk EC behaviors can only be reached from a few initial EC behaviors, and also that the Tip EC behavior prevents direct full EndMT. Tip ECs may undergo indirect full EndMT only by previously transforming into nonphalanx, nonstalk, and nontip ECs or into mesenchymal stalk cells. Therefore, our model constitutes a theoretical framework that enables hypothesis generation, and illuminates and restricts the possible paths for future experimental EndMT research and the pharmacological control of EndMT.

## Data Availability Statement

The datasets generated for this study can be found in the GitHub https://github.com/NathanWeinstein/EndMT.

## Author Contributions

NW, LM, and EÁ-B planned the research, wrote the article, and analyzed and discussed the results. NW reviewed the literature, composed the model, wrote the update rule, wrote the required scripts and made the tables and figures. NW and LM carried out the simulations. LM and EÁ-B obtained funding for this project.

## Funding

This work was funded by the UNAM-DGAPA postdoctoral scholarship to NW; grant UNAM-DGAPA-PAPIIT IN200918 to LM; and grants CONACYT CB 2014-240180-B, CONACYT PN 2015-01-687, and UNAM-DGAPA-PAPIIT IN208517 to EÁ-B.

## Conflict of Interest

The authors declare that the research was conducted in the absence of any commercial or financial relationships that could be construed as a potential conflict of interest.
