# Supplementary material for: A Computational Model of the Endothelial to Mesenchymal Transition
Source: Front Genet. 2020 Mar 12;11:40. doi: 10.3389/fgene.2020.00040 (PMC7080988; doi:10.3389/fgene.2020.00040)
Supplement: Supplementary file 1 [file DataSheet_1.pdf]

Supplementary Material: A Computational Model of the Endothelial to Mesenchymal Transition

1 SUPPLEMENTARY DATA

The datasets generated for this study can be found in the GitHub https://github.com/NathanWeinstein/EndMT.

2 SUPPLEMENTARY TABLES AND FIGURES

Number	Feedback circuit	Sign
1	FLI1	Positive
2	GATA2	Positive
3	LEF1	Positive
4	SNAI2	Positive
5	VEGFA	Positive
6	ZEB2 ETS1	Positive
7	ETS1 VEGFR2	Positive
8	SMAD2 AP1 ETS1 NRP1	Positive
9	NRARP NOTCH	Negative
10	SMAD6 SMAD1	Negative
11	SNAI1	Negative
12	SNAI1 SNAI2	Not Functional
13	NRP1 SMAD2 SNAI1 ZEB2 ETS1	Not Functional
14	SNAI1 ZEB2 ETS1 VEGFR2 STAT3	Not Functional
15	NRP1 SMAD2 SNAI1 ZEB2 ETS1 VEGFR2 STAT3 VEGFA	Not Functional
16	VEGFR2 STAT3 VEGFA	Not Functional
17	NRP1 SMAD2 SNAI1 ZEB2 ETS1 VEGFR2 GATA2	Not Functional
18	SNAI1 ZEB2 ETS1 VEGFR2 GATA2 SNAI2	Not Functional
19	VEGFR2 GATA2 FLI1	Not Functional
20	GATA2 FLI1	Not Functional
21	SMAD2 SNAI1 ZEB2 ETS1 VEGFR2 GATA2 FLI1 SMAD6	Not Functional
22	VEGFR2 GATA2	Not Functional
23	SMAD2 SNAI1 ZEB2 ETS1 VEGFR2 GATA2 SMAD6	Not Functional
24	SNAI1 VEGFR2 STAT3	Not Functional
25	NRP1 SMAD2 SNAI1 VEGFR2 STAT3 VEGFA	Not Functional
26	NRP1 SMAD2 SNAI1 VEGFR2 ETS1	Not Functional
27	NRP1 SMAD2 SNAI1 VEGFR2 GATA2	Not Functional
28	SNAI1 VEGFR2 GATA2 SNAI2	Not Functional
29	SMAD2 SNAI1 VEGFR2 GATA2 FLI1 SMAD6	Not Functional
30	SMAD2 SNAI1 VEGFR2 GATA2 SMAD6	Not Functional
31	NRP1 SMAD2 SNAI2 SNAI1 ZEB2 ETS1	Not Functional

32	NRP1 SMAD2 SNAI2 SNAI1 ZEB2 ETS1 VEGFR2 STAT3 VEGFA	Not Functional
33	NRP1 SMAD2 SNAI2 SNAI1 ZEB2 ETS1 VEGFR2 GATA2	Not Functional
34	SMAD2 SNAI2 SNAI1 ZEB2 ETS1 VEGFR2 GATA2 FLI1 SMAD6	Not Functional
35	SMAD2 SNAI2 SNAI1 ZEB2 ETS1 VEGFR2 GATA2 SMAD6	Not Functional
36	NRP1 SMAD2 SNAI2 SNAI1 VEGFR2 STAT3 VEGFA	Not Functional
37	NRP1 SMAD2 SNAI2 SNAI1 VEGFR2 ETS1	Not Functional
38	NRP1 SMAD2 SNAI2 SNAI1 VEGFR2 GATA2	Not Functional
39	SMAD2 SNAI2 SNAI1 VEGFR2 GATA2 FLI1 SMAD6	Not Functional
40	SMAD2 SNAI2 SNAI1 VEGFR2 GATA2 SMAD6	Not Functional
41	NRP1 SMAD2 ZEB2 ETS1	Not Functional
42	NRP1 SMAD2 ZEB2 ETS1 VEGFR2 STAT3 VEGFA	Not Functional
43	NRP1 SMAD2 ZEB2 ETS1 VEGFR2 GATA2	Not Functional
44	SMAD2 ZEB2 ETS1 VEGFR2 GATA2 FLI1 SMAD6	Not Functional
45	SMAD2 ZEB2 ETS1 VEGFR2 GATA2 SMAD6	Not Functional
46	NRP1 SMAD2 LEF1 SNAI2 SNAI1 ZEB2 ETS1	Not Functional
47	NRP1 SMAD2 LEF1 SNAI2 SNAI1 ZEB2 ETS1 VEGFR2 STAT3 VEGFA	Not Functional
48	NRP1 SMAD2 LEF1 SNAI2 SNAI1 ZEB2 ETS1 VEGFR2 GATA2	Not Functional
49	SMAD2 LEF1 SNAI2 SNAI1 ZEB2 ETS1 VEGFR2 GATA2 FLI1 SMAD6	Not Functional
50	SMAD2 LEF1 SNAI2 SNAI1 ZEB2 ETS1 VEGFR2 GATA2 SMAD6	Not Functional
51	NRP1 SMAD2 LEF1 SNAI2 SNAI1 VEGFR2 STAT3 VEGFA	Not Functional
52	NRP1 SMAD2 LEF1 SNAI2 SNAI1 VEGFR2 ETS1	Not Functional
53	NRP1 SMAD2 LEF1 SNAI2 SNAI1 VEGFR2 GATA2	Not Functional
54	SMAD2 LEF1 SNAI2 SNAI1 VEGFR2 GATA2 FLI1 SMAD6	Not Functional
55	SMAD2 LEF1 SNAI2 SNAI1 VEGFR2 GATA2 SMAD6	Not Functional
56	NRP1 SMAD2 AP1 SNAI1 ZEB2 ETS1	Not Functional
57	NRP1 SMAD2 AP1 SNAI1 ZEB2 ETS1 VEGFR2 STAT3 VEGFA	Not Functional
58	NRP1 SMAD2 AP1 SNAI1 ZEB2 ETS1 VEGFR2 GATA2	Not Functional
59	SMAD2 AP1 SNAI1 ZEB2 ETS1 VEGFR2 GATA2 FLI1 SMAD6	Not Functional
60	SMAD2 AP1 SNAI1 ZEB2 ETS1 VEGFR2 GATA2 SMAD6	Not Functional
61	NRP1 SMAD2 AP1 SNAI1 VEGFR2 STAT3 VEGFA	Not Functional
62	NRP1 SMAD2 AP1 SNAI1 VEGFR2 ETS1	Not Functional
63	NRP1 SMAD2 AP1 SNAI1 VEGFR2 GATA2	Not Functional
64	SMAD2 AP1 SNAI1 VEGFR2 GATA2 FLI1 SMAD6	Not Functional
65	SMAD2 AP1 SNAI1 VEGFR2 GATA2 SMAD6	Not Functional
66	NRP1 SMAD2 AP1 ZEB2 ETS1	Not Functional
67	NRP1 SMAD2 AP1 ZEB2 ETS1 VEGFR2 STAT3 VEGFA	Not Functional
68	NRP1 SMAD2 AP1 ZEB2 ETS1 VEGFR2 GATA2	Not Functional
69	SMAD2 AP1 ZEB2 ETS1 VEGFR2 GATA2 FLI1 SMAD6	Not Functional
70	SMAD2 AP1 ZEB2 ETS1 VEGFR2 GATA2 SMAD6	Not Functional
71	NRP1 SMAD2 AP1 ETS1 VEGFR2 STAT3 VEGFA	Not Functional
72	NRP1 SMAD2 AP1 ETS1 VEGFR2 GATA2	Not Functional
73	SMAD2 AP1 ETS1 VEGFR2 GATA2 FLI1 SMAD6	Not Functional
74	SMAD2 AP1 ETS1 VEGFR2 GATA2 SMAD6	Not Functional

Table S1: The positive, negative and non-functional feedback circuits present in our model.

Molecule	Loss of function	Simulated loss of function	Gain of function	Simulated gain of function
AP1	c-Jun siRNA reduced SNAI1 expression in mesenchymal cells during EMT (Nguyen et al., 2013).	SNAI1 is not expressed. SNAI1 inhibits its own activity, and is not expressed in the wild type either.	—	Loss of non-mesenchymal stalk cells
CTNNB	Endothelial-specific deletion causes excessive vessel regression (Reis and Liebner, 2013)	Loss of non-mesenchymal stalk cells.	Activates the transcription of SNAI2, TWIST1 and ZEB1 during EMT (Howe et al., 2003; Menezes, 2014)	Causes the loss of phalanx, and non-phalanx, non-tip, and non-stalk ECs. More fixed and cyclic patterns of molecular activation express SNAI2, TWIST1 and ZEB1.
DLL4	Larger, dysfunctional vascular networks that cause poor perfusion (Alabi et al., 2018).	Wild type.	Promotes arterial EC specification. Reduces EC response to VEGF, decreases EC proliferation, vessel density, and perfusion (Alabi et al., 2018).	Loss of phalanx cells and tip cells
ETS1	Some effects masked by functional redundancy with ETS2. Inhibits EC migration and VEGF-induced EC proliferation, and inhibits retinal angiogenesis (Craig and Sumanas, 2016)	Loss of tip cells.	Induces EC apoptosis in the absence of VEGFA, up-regulates MMP-1, MMP-3 and MMP-9 expression, increases EC invasiveness (Craig and Sumanas, 2016)	Loss of non-mesenchymal stalk cells

FGF2	Delayed wound healing and suppressed VEGFR2 expression (Yang et al., 2015). Mouse lymphatic ECs undergo EndMT (Ichise et al., 2014).	Reduces the fraction of fixed or cyclic patterns of molecular activation that express VEGFR2	Promotes angiogenesis, and EC proliferation (Yang et al., 2015). Suppresses EndMT in mouse lymphatic ECs (Ichise et al., 2014).	Reduces the number of non-endothelial and mesenchymal attractors from 48 in the wild type to 24.
FLI1	Lethal in mice during embryonic day 12.0 due to hemorrhage caused by excessive EC death, affected endocardial cushion formation and diminished VE cadherin, PECAM1, and TIE2 expression (Abedin et al., 2014). Triggers EndMT (Nagai et al., 2018)	Loss of all ECs. Induces EndMT.	Augmented FLI1 and TIE2 expression (Abedin et al., 2014). Increased GATA2 and VEGFR2 expression (Liu et al., 2008)	Loss of non-endothlial cells. Increases VEGFR2 and GATA2 expression
GATA2	In mice, causes embryonic lethality between embryonic days 9.5–11.5, and triggers EndMT (Kanki et al., 2011). Inhibits VEGFA-induced EC migration and angiogenesis and lymphangiogenesis, downregulates the expression of NRP1, VEGFR1, VEGFR2 and VEGFR3 (Coma et al., 2013).	Loss of all ECs. Induces EndMT, inhibits VEGFR2 expression.	–	Loss of non-endothlial cells

HIF1α	Averts hypoxia-mediated EndMT also prevents hypoxia-mediated SNAI1 and TWIST1 expression (Xu et al., 2015; Yang and Wu, 2008).	Decreases the expression of TWIST1, also reduces the fraction of mesenchymal attractors from 0.567 to 0.429.	Induces EndMT, activates the expression of SNAI1 (Xu et al., 2015)	Increases the fraction of mesenchymal attractors from 0.567 to 1.
LEF1	Reduced blood vessel density (Pate et al., 2014).	Wild type.	Induces EMT (Kobayashi and Ozawa, 2018)	Loss of phalanx cells. Increases the fraction of mesenchymal attractors from 0.567 to 0.626.
NFκB	Inhibited PDGF-induced SNAI1 expression (Liu et al., 2018)	SNAI1 is not active in any attractor.	Induced EndMT, augmented EC invasiveness, and increased the expression of SNAI1, ZEB2, TGFβ1, and ACTA2 (Mahler et al., 2013; Julien et al., 2007).	Increases the expression of ZEB2. Increases the fraction of mesenchymal attractors from 0.567 to 0.626.
NOTCH1	EC-specific loss is lethal (Limbourg et al., 2005). ECs preferentially assume tip-cell characteristics (Hellström et al., 2007), and augmented NRP1 expression (Aspalter et al., 2015). Bicuspid aortic valve and aortic valve calcification (Garg et al., 2005).	Increases the fraction of tip attractors from 0.164 to 0.304. Increases the expression of NRP1.	ECs preferentially assume Stalk cell characteristics over Tip cells (Hellström et al., 2007). Increases AKT2 expression, and decreases AKT1 expression leading to GSK3β phosphorylation which prevents SNAI1 degradation (Frías et al., 2015). Upregulates the expression of SNAI2 (Niessen et al., 2008).	Loss of Tip and Phalanx cells. Increases SNAI1 expression from 0 to 0.04. Increases the fraction of stalk attractors from 0.239 to 0.414.

NRP1	In mice, causes lethal defects in the cardiovascular system. Prevents EC proliferation and angiogenesis (Oh et al., 2002). Augments SMAD2 phosphorylation (Aspalter et al., 2015). Inhibits TGFβ1-induced EndMT and pSMAD2 signaling (Matkar et al., 2016).	Loss of tip cells. Completely inhibits SMAD2 activity.	Dowregulates SMAD2 phosphorylation (Aspalter et al., 2015). Induces TGFβ1-mediated EndMT and up-regulates the expression of TGFBR1 and TGFBR2 (Matkar et al., 2016).	Loss of Stalk cells, Phalanx cells, and mesenchymal ECs that are neither phalanx, tip or stalk cells. Strongly upregulates SMAD2 and slightly downregulates SMAD1. Increases the fraction of mesenchymal attractors from 0.567 in the wild type to 0.669
NRARP	Causes blood vessel regression, delayed vascularization, and decreases blood vessel density (Phng et al., 2009),	Wild type.	Inhibits the activity of NOTCH (Lamar et al., 2001).	Loss of phalanx cells. Completely prevents NOTCH activity.
PDGF_AB	Inhibits EndMT in glioblastoma multiforme-associated ECs (Liu et al., 2018). Prevents epicardial EMT (von Gise and Pu, 2012)	Reduces the fraction of mesenchymal attractors from 0.567 in the wild type to 0.520	Induces EndMT, activates NFkB which induces the expression of SNAI1 (Liu et al., 2018).	Loss of phalanx cells. Increaces the fraction of mesenchymal attractors from 0.567 in the wild type to 0.626. Upregulates NFkB.
SMAD1	Lethal in mice, EC specific knock out develops PAH (Jin et al., 2014). DN-SMAD4 (cofactor of both SMAD1 and SMAD2) prevents TGFβ2-mediated SNAI1 expression (Medici et al., 2011)and endocardial EndMT (von Gise and Pu, 2012).	No SNAI1 activity like the wild type	NA	Wild type

SMAD2	Lethal in mice (Jin et al., 2014). DN-SMAD4 (cofactor of both SMAD1 and SMAD2) prevents TGFβ2-mediated SNAI1 expression (Medici et al., 2011)and endocardial EndMT (von Gise and Pu, 2012).	No SNAI1 activity, like the wild type.	NA	Loss of phalanx and non-mesenchymal stalk cells.
SMAD6	Sometimes lethal, excess mesenchymal cells in the cardiac valves, outflow tract septation defects, arterial ossification and elevated blood pressure (Galvin et al., 2000). Increased BMP6-induced nuclear pSMAD1/5 (Mouillesseaux et al., 2016).	Increases the fraction of fixed or cyclic patterns of molecular activation patterns with active SMAD1 from 0.263 to 0.334.	Decreased BMP6-induced nuclear pSMAD1/5 (Mouillesseaux et al., 2016).	Prevents SMAD1 activation.
SNAI1	Inhibits angiogenic sprouting including retinal neovascularization, impaires VEGF-induced cell morphological change and lamellipodia formation, reduces EC motility and invasion (Sun et al., 2018).	Wild type.	Increases EC motility and invasion (Sun et al., 2018). Is sufficient to trigger EndMT especially in arterial ECs, in particular decreases VE-cadherin expression (Pinto et al., 2018).	Loss of Phalanx and Stalk cells. Increases the fraction of mesencymal attractors from 0.567 to 0.769.

SNAI2	In mice (E9.5), leads to impaired cardiac cushion development, however, increased SNAI1 expression during E10.5 restores cardiac cushion EndMT Niessen et al. (2008). Inhibits sprouting angiogenesis and lumen formation, inhibits MT1-MMP and JAG1 expression, reduces MMP2 and MMP9 activity (Welch-Reardon et al., 2015).	Loss of stalk cells. Prevents EndMT due to the loss of all mesenchymal attractors.	Promotes EC sprouting, sprout detachment (Welch-Reardon et al., 2015). Repressed the expression of VE-cadherin, PECAM1, and TIE2 (Niessen et al., 2008).	Loss of phalanx cells. Increases the fraction of mesenchymal attractors from 0.567 to 0.597.
STAT3	Inhibits SNAI1 expression (Saitoh et al., 2016). Impairs VEGF-mediated promotion of EC survival (Chen et al., 2008).	No SNAI1 activity.	Induces SNAI1 expression (Saitoh et al., 2016)	Wild type, no SNAI1 activity.

TGFβ	In mice, loss of TGF-β2 inhibits EndMT-mediated cardiac development, while loss of TGF-β1 or TGF-β3 has no significant effects on EndMT and heart development (Medici et al., 2011).	Inhibits EndMT.	In human cutaneous microvascular endothelial cells, exposure to TGF-β2 increases the level of phosphorylation of ERK1/2, AKT, and p38 MAPK and leads to SNAI1-mediated EndMT (Medici et al., 2011). However, the the anatomical origin of ECs influences their ability to undergo TGFβ2-mediated EndMT (Ursoli Ferreira et al., 2019),	Induces EndMT. No SNAI1 activity , like thw wild type.
TGFβR	ALK5 loss is lethal in mice, with defective yolk sac vascular development (Jin et al., 2014). LoeysDietz syndrome (LDS) (Pardali and Ten Dijke, 2012). Prevents EndMT caused by loss of FGF signaling (Chen et al., 2012).	Reduces the fraction of mesenchimal attracors from 0.567 to 0.565	Constitutive TGF-βR1(ALK5) activity increases the expression of smooth muscle cell and mesenchymal cell markers and increases SMAD2 phosphorylation (Chen et al., 2012).	Increases the fraction of mesenchymal attractors from 0.567 to 0.570. Increases SMAD2 activity from 0.052 to 0.155.
TWIST1	Inhibits hypoxia-induced EndMT (Mammoto et al., 2018).	Loss of mesenchymal cells. Prevents EndMT.	Increases TGF-βR2 expression and SMAD2 phosphorylation, and induces EndMT in cultured human pulmonary arterial endothelial (HPAE) cells (Mammoto et al., 2018).	Loss of phalanx cells, and non-mesenchymal tip cells. Increases the fraction of mesencymal attractors from 0.567 to 0.737. Upregulates SMAD2, not TGFBR.

VEGFA	Lethal with impaired angiogenesis (Ferrara et al., 1996), EC specific deletion is also lethal (Guangqi et al., 2012). Inhibits the expression of VEGFR2, TIE2 and VE-cadherin (Guangqi et al., 2012).	Loss of tip cells. Completely inhibits VEGFR2 activity.	Inhibits TGFβ2-induced EndMT (Paruchuri et al., 2006), and it is lethal in mice due to abnormal heart development (Guangqi et al., 2012).	Loss of phalanx, stalk, and non-endothelial mesenchymal cells. Increases the fraction of mesenchymal attractors from 0.567 to 0.732
VEGFR2	Lethal in mice, causes defective blood vessel organization (Sakurai et al., 2005).	Wild type.	Causes vascular tumours (Carmeliet and Jain, 2011).	Loss of non-endothlial and non-mesenchymal cells, non-mesenchymal stalk cells, and non-endothelial mesenchymal cells.
WNT5b	Hindered lymphangiogenesis *in vitro* and EndMT-mediated lymph node metastasis *in vivo* (Wang et al., 2017b).	Reduces the fracion of mesenchymal attractors from 0.567 to 0.324	Induced partial EndoMT in lymphatic ECs, increased the protein levels of SNAI1 and SNAI2 (Wang et al., 2017b).	All attractors are mesenchymal.
WNT7a	Loss of the similar ligand WNT1 leads to osteogenesis imperfecta, osteoporosis, recurrent fractures Keupp et al. (2013)	Loss of non-mesenchymal stalk cells.	Proangiogenic, increases EC migration (Pahnke et al., 2016). Activates TWIST1 expression (Howe et al., 2003).	Loss of phalanx cells, non-mesenchymal tip cells, and non-mesenchymal, non-phalanx, non-tip, and non-stalk ECs. Induces TWIST1 expression.

ZEB1	Endothelial corneal dystrophies (Chung et al., 2014). Leads to ectopic E-cadherin expression and inhibits the expression of mesenchymal genes (Sanchez-Tillo et al., 2010).	Prevents EndMT, no mesenchymal attractors.	Induces EMT (Sanchez-Tillo et al., 2010).	Wild type.
ZEB2	Severe neurodevelopmental defects and cardiovascular malformations (Epifanova et al., 2018).	Prevents EndMT, no mesenchymal attractors.	Inhibits high glucose-mediated EC apoptosis (Wang et al., 2017a). Causes EMT (DaSilva-Arnold et al., 2018).	Loss of non-mesenchymal stalk cells. Induces EndMT increases the fraction of mesenchymal attractors from 0.567 to 0.783

Table S2: The specific effects of single gain and loss-of-function mutations.

REFERENCES

Abedin, M. J., Nguyen, A., Jiang, N., Perry, C. E., Shelton, J. M., Watson, D. K., et al. (2014). Fli1 acts downstream of Etv2 to govern cell survival and vascular homeostasis via positive autoregulation. *Circulation research* , CIRCRESAHA–113doi:10.1161/CIRCRESAHA.1134303145

Alabi, R. O., Farber, G., and Blobel, C. P. (2018). Intriguing roles for endothelial ADAM10/Notch signaling in the development of organ-specific vascular beds. *Physiological reviews* 98, 2025–2061. doi:10.1152/physrev.00029.2017

Aspalter, I. M., Gordon, E., Dubrac, A., Ragab, A., Narloch, J., Vizán, P., et al. (2015). Alk1 and alk5 inhibition by nrp1 controls vascular sprouting downstream of notch. *Nature communications* 6, 7264. doi:10.1038/ncomms8264

Carmeliet, P. and Jain, R. K. (2011). Molecular mechanisms and clinical applications of angiogenesis. *Nature* 473, 298–307. doi:10.1038/nature10144

Chen, P.-Y., Qin, L., Barnes, C., Charisse, K., Yi, T., Zhang, X., et al. (2012). FGF regulates TGF-β signaling and endothelial-to-mesenchymal transition via control of let-7 mirna expression. *Cell reports* 2, 1684–1696. doi:10.1016/j.celrep.2012.10.021

Chen, S.-H., Murphy, D., Lassoued, W., Thurston, G., Feldman, M. D., and Lee, W. M. (2008). Activated STAT3 is a mediator and biomarker of VEGF endothelial activation. *Cancer biology & therapy* 7, 1994–2003. doi:10.4161/cbt.7.12.6967

Chung, D.-W. D., Frausto, R. F., Ann, L. B., Jang, M. S., and Aldave, A. J. (2014). Functional impact of zeb1 mutations associated with posterior polymorphous and fuchs' endothelial corneal dystrophies. *Investigative ophthalmology & visual science* 55, 6159–6166. doi:10.1167/iovs.14-15247

Coma, S., Allard-Ratick, M., Akino, T., van Meeteren, L. A., Mammoto, A., and Klagsbrun, M. (2013). Gata2 and lmo2 control angiogenesis and lymphangiogenesis via direct transcriptional regulation of neuropilin-2. *Angiogenesis* 16, 939–952. doi:10.1007/s10456-013-9370-9

Craig, M. P. and Sumanas, S. (2016). Ets transcription factors in embryonic vascular development. *Angiogenesis* 19, 275–285. doi:10.1007/s10456-016-9511-z

DaSilva-Arnold, S. C., Kuo, C.-Y., Davra, V., Remache, Y., Kim, P. C., Fisher, J. P., et al. (2018). Zeb2, a master regulator of the epithelial–mesenchymal transition, mediates trophoblast differentiation. *MHR: Basic science of reproductive medicine* 25, 61–75. doi:10.1093/molehr/gay053

Epifanova, E., Babaev, A., Newman, A., and Tarabykin, V. (2018). Role of zeb2/sip1 in neuronal development. *Brain research* 1705, 24–31. doi:10.1016/j.brainres.2018.09.034

Ferrara, N., Carver-Moore, K., Chen, H., Dowd, M., et al. (1996). Heterozygous embryonic lethality induced by targeted inactivation of the VEGF gene. *Nature* 380, 439. doi:10.1038/380439a0

Frías, A., Lambies, G., Viñas-Castells, R., Martínez-Guillamon, C., Dave, N., de Herreros, A. G., et al. (2015). A switch in akt isoforms is required for notch-induced snail1 expression and protection from cell death. *Molecular and cellular biology* , MCB–01074doi:10.1128/MCB.01074-15

Galvin, K. M., Donovan, M. J., Lynch, C. A., Meyer, R. I., Paul, R. J., Lorenz, J. N., et al. (2000). A role for smad6 in development and homeostasis of the cardiovascular system. *Nature genetics* 24, 171. doi:10.1038/72835

Garg, V., Muth, A. N., Ransom, J. F., Schluterman, M. K., Barnes, R., King, I. N., et al. (2005). Mutations in notch1 cause aortic valve disease. *Nature* 437, 270. doi:10.1038/nature03940

Guangqi, E., Cao, Y., Bhattacharya, S., Dutta, S., Wang, E., and Mukhopadhyay, D. (2012). Endogenous vascular endothelial growth factor-A (VEGF-A) maintains endothelial cell homeostasis by regulating VEGF receptor-2 transcription. *Journal of Biological Chemistry* 287, 3029–3041. doi:10.1074/jbc.M111.29398

Hellström, M., Phng, L.-K., Hofmann, J. J., Wallgard, E., Coultas, L., Lindblom, P., et al. (2007). Dll4 signalling through Notch1 regulates formation of tip cells during angiogenesis. *Nature* 445, 776–780. doi:10.1038/nature05571

Howe, L. R., Watanabe, O., Leonard, J., and Brown, A. M. (2003). Twist is up-regulated in response to wnt1 and inhibits mouse mammary cell differentiation. *Cancer research* 63, 1906–1913

Ichise, T., Yoshida, N., and Ichise, H. (2014). FGF2-induced Ras–MAPK signalling maintains lymphatic endothelial cell identity by upregulating endothelial-cell-specific gene expression and suppressing tgfβ signalling through smad2. *J Cell Sci* 127, 845–857. doi:10.1242/jcs.137836

Jin, Y., Kaluza, D., and Jakobsson, L. (2014). VEGF, Notch and TGFβ/BMPs in regulation of sprouting angiogenesis and vascular patterning. *Biochemical Society Transactions* 42, 1576–1583. doi:10.1042/BST20140231

Julien, S., Puig, I., Caretti, E., Bonaventure, J., Nelles, L., Van Roy, F., et al. (2007). Activation of nf-κb by akt upregulates snail expression and induces epithelium mesenchyme transition. *Oncogene* 26, 7445

Kanki, Y., Kohro, T., Jiang, S., Tsutsumi, S., Mimura, I., Suehiro, J.-i., et al. (2011). Epigenetically coordinated GATA2 binding is necessary for endothelium-specific endomucin expression. *The EMBO journal* 30, 2582–2595. doi:10.1038/emboj.2011.173

Keupp, K., Beleggia, F., Kayserili, H., Barnes, A. M., Steiner, M., Semler, O., et al. (2013). Mutations in wnt1 cause different forms of bone fragility. *The American Journal of Human Genetics* 92, 565–574. doi:10.1016/j.ajhg.2013.02.010

Kobayashi, W. and Ozawa, M. (2018). The epithelial-mesenchymal transition induced by transcription factor LEF-1 is independent of β-catenin. *Biochemistry and biophysics reports* 15, 13–18. doi:10.1016/j.bbrep.2018.06.003

Lamar, E., Deblandre, G., Wettstein, D., Gawantka, V., Pollet, N., Niehrs, C., et al. (2001). Nrarp is a novel intracellular component of the Notch signaling pathway. *Genes & development* 15, 1885–1899. doi:10.1101/gad.908101

Limbourg, F. P., Takeshita, K., Radtke, F., Bronson, R. T., Chin, M. T., and Liao, J. K. (2005). Essential role of endothelial Notch1 in angiogenesis. *Circulation* 111, 1826–1832. doi:10.1161/01.CIR.0000160870.93058.DD

Liu, F., Walmsley, M., Rodaway, A., and Patient, R. (2008). Fli1 acts at the top of the transcriptional network driving blood and endothelial development. *Current Biology* 18, 1234–1240. doi:10.1016/j.cub.2008.07.048

Liu, T., Ma, W., Xu, H., Huang, M., Zhang, D., He, Z., et al. (2018). PDGF-mediated mesenchymal transformation renders endothelial resistance to anti-VEGF treatment in glioblastoma. *Nature communications* 9, 3439. doi:10.1038/s41467-018-05982-z

Mahler, G. J., Farrar, E. J., and Butcher, J. T. (2013). Inflammatory cytokines promote mesenchymal transformation in embryonic and adult valve endothelial cells. *Arteriosclerosis, thrombosis, and vascular biology* 33, 121–130. doi:10.1161/ATVBAHA.112.300504

Mammoto, T., Muyleart, M., Konduri, G. G., and Mammoto, A. (2018). Twist1 in hypoxia-induced pulmonary hypertension through transforming growth factor-β–Smad signaling. *American journal of respiratory cell and molecular biology* 58, 194–207. doi:10.1165/rcmb.2016-0323OC

Matkar, P. N., Singh, K. K., Rudenko, D., Kim, Y. J., Kuliszewski, M. A., Prud'homme, G. J., et al. (2016). Novel regulatory role of neuropilin-1 in endothelial-to-mesenchymal transition and fibrosis in pancreatic ductal adenocarcinoma. *Oncotarget* 7, 69489. doi:10.18632/oncotarget.11060

Medici, D., Potenta, S., and Kalluri, R. (2011). Transforming growth factor-β2 promotes Snail-mediated endothelial–mesenchymal transition through convergence of Smad-dependent and Smad-independent signalling. *Biochemical Journal* 437, 515–520. doi:10.1042/BJ20101500

Menezes, M. E. (2014). The wnt/β-catenin signaling pathway in epithelial mesenchymal transition. *J Postdoctoral Res* 1, 12

Mouillesseaux, K. P., Wiley, D. S., Saunders, L. M., Wylie, L. A., Kushner, E. J., Chong, D. C., et al. (2016). Notch regulates bmp responsiveness and lateral branching in vessel networks via smad6. *Nature communications* 7, 13247. doi:10.1038/ncomms13247

Nagai, N., Ohguchi, H., Nakaki, R., Matsumura, Y., Kanki, Y., Sakai, J., et al. (2018). Downregulation of ERG and FLI1 expression in endothelial cells triggers endothelial-to-mesenchymal transition. *PLoS genetics* 14, e1007826. doi:10.1371/journal.pgen.1007826

Nguyen, P., Tsunematsu, T., Yanagisawa, S., Kudo, Y., Miyauchi, M., Kamata, N., et al. (2013). The fgfr1 inhibitor pd173074 induces mesenchymal–epithelial transition through the transcription factor ap-1. *British journal of cancer* 109, 2248. doi:10.1038/bjc.2013.550

Niessen, K., Fu, Y., Chang, L., Hoodless, P. A., McFadden, D., and Karsan, A. (2008). Slug is a direct notch target required for initiation of cardiac cushion cellularization. *The Journal of cell biology* 182, 315–325. doi:10.1083/jcb.200710067

Oh, H., Takagi, H., Otani, A., Koyama, S., Kemmochi, S., Uemura, A., et al. (2002). Selective induction of neuropilin-1 by vascular endothelial growth factor (vegf): a mechanism contributing to vegf-induced angiogenesis. *Proceedings of the National Academy of Sciences* 99, 383–388. doi:10.1073/pnas.012074399

Pahnke, A., Conant, G., Huyer, L. D., Zhao, Y., Feric, N., and Radisic, M. (2016). The role of wnt regulation in heart development, cardiac repair and disease: A tissue engineering perspective. *Biochemical and biophysical research communications* 473, 698–703. doi:10.1016/j.bbrc.2015.11.060

Pardali, E. and Ten Dijke, P. (2012). TGFbeta signaling and cardiovascular diseases. *Int J Biol Sci* 8, 195–213. doi:10.7150/ijbs.3805

Paruchuri, S., Yang, J.-H., Aikawa, E., Melero-Martin, J. M., Khan, Z. A., Loukogeorgakis, S., et al. (2006). Human pulmonary valve progenitor cells exhibit endothelial/mesenchymal plasticity in response to vascular endothelial growth factor-a and transforming growth factor-β2. *Circulation research* 99, 861–869. doi:10.1161/01.RES.0000245188.41002.2c

Pate, K. T., Stringari, C., Sprowl-Tanio, S., Wang, K., TeSlaa, T., Hoverter, N. P., et al. (2014). Wnt signaling directs a metabolic program of glycolysis and angiogenesis in colon cancer. *The EMBO journal* 33, 1454–1473. doi:10.15252/embj.201488598

Phng, L.-K., Potente, M., Leslie, J. D., Babbage, J., Nyqvist, D., Lobov, I., et al. (2009). Nrarp coordinates endothelial Notch and Wnt signaling to control vessel density in angiogenesis. *Developmental cell* 16, 70–82. doi:10.1016/j.devcel.2008.12.009

Pinto, M. T., Melo, F. U. F., Malta, T. M., Rodrigues, E. S., Placa, J. R., Silva Jr, W. A., et al. (2018). Endothelial cells from different anatomical origin have distinct responses during snail/tgf-β2-mediated endothelial-mesenchymal transition. *American journal of translational research* 10, 4065

Reis, M. and Liebner, S. (2013). Wnt signaling in the vasculature. *Experimental cell research* 319, 1317–1323. doi:10.1016/j.yexcr.2012.12.023

Saitoh, M., Endo, K., Furuya, S., Minami, M., Fukasawa, A., Imamura, T., et al. (2016). Stat3 integrates cooperative ras and tgf-β signals that induce snail expression. *Oncogene* 35, 1049. doi:10.1038/onc.2015.161

Sakurai, Y., Ohgimoto, K., Kataoka, Y., Yoshida, N., and Shibuya, M. (2005). Essential role of Flk-1 (VEGF receptor 2) tyrosine residue 1173 in vasculogenesis in mice. *Proceedings of the National Academy of Sciences of the United States of America* 102, 1076–1081. doi:10.1073/pnas.0404984102

Sanchez-Tillo, E., Lazaro, A., Torrent, R., Cuatrecasas, M., Vaquero, E., Castells, A., et al. (2010). Zeb1 represses e-cadherin and induces an emt by recruiting the swi/snf chromatin-remodeling protein brg1. *Oncogene* 29, 3490. doi:10.1038/onc.2010.102

Sun, J.-X., Chang, T.-F., Li, M.-H., Sun, L.-J., Yan, X.-C., Yang, Z.-Y., et al. (2018). Snai1, an endothelial–mesenchymal transition transcription factor, promotes the early phase of ocular neovascularization. *Angiogenesis* 21, 635–652. doi:10.1007/s10456-018-9614-9

Ursoli Ferreira, F., Eduardo Botelho Souza, L., Hassibe Thomé, C., Tomazini Pinto, M., Origassa, C., Salustiano, S., et al. (2019). Endothelial Cells Tissue-Specific Origins Affects Their Responsiveness to TGF-β2 during Endothelial-to-Mesenchymal Transition. *International journal of molecular sciences* 20, 458. doi:10.3390/ijms20030458

von Gise, A. and Pu, W. T. (2012). Endocardial and epicardial epithelial to mesenchymal transitions in heart development and disease. *Circulation research* 110, 1628–1645. doi:10.1161/CIRCRESAHA.111.259960

Wang, L.-J., Wu, Z.-H., Zheng, X.-T., Long, J.-Y., Dong, Y.-M., and Fang, X. (2017a). Zinc finger e-box binding protein 2 (zeb2) suppress apoptosis of vascular endothelial cells induced by high glucose through mitogen-activated protein kinases (mapk) pathway activation. *Medical science monitor: international medical journal of experimental and clinical research* 23, 2590. doi:10.12659/MSM.904678

Wang, S., Chang, J., Hsiao, J., Yen, Y., Jiang, S., Liu, S., et al. (2017b). Tumour cell-derived wnt5b modulates in vitro lymphangiogenesis via induction of partial endothelial-mesenchymal transition of lymphatic endothelial cells. *Oncogene* 36, 1503. doi:10.1038/onc.2016.317

Welch-Reardon, K. M., Wu, N., and Hughes, C. C. (2015). A role for partial endothelial–mesenchymal transitions in angiogenesis? *Arteriosclerosis, thrombosis, and vascular biology* 35, 303–308. doi:10.1161/ATVBAHA.114.303220

Xu, X., Tan, X., Tampe, B., Sanchez, E., Zeisberg, M., and Zeisberg, E. M. (2015). Snail is a direct target of hypoxia-inducible factor 1α (HIF1α) in hypoxia-induced endothelial to mesenchymal transition of human coronary endothelial cells. *Journal of Biological Chemistry* 290, 16653–16664. doi:10.1074/jbc.M115.636944

Yang, M.-H. and Wu, K.-J. (2008). Twist activation by hypoxia inducible factor-1 (HIF-1): implications in metastasis and development. *Cell cycle* 7, 2090–2096. doi:10.4161/cc.7.14.6324

Yang, X., Liaw, L., Prudovsky, I., Brooks, P. C., Vary, C., Oxburgh, L., et al. (2015). Fibroblast growth factor signaling in the vasculature. *Current atherosclerosis reports* 17, 31. doi:10.1007/s11883-015-0509-6
